# The ADIPO-CARE Plan: A Comprehensive Implementation Framework for the Diagnosis and Management of People Living with Obesity—Experts’ Proposal

**DOI:** 10.3390/nu18213662

**Published:** 2026-11-04

**Authors:** Stanisław Surma, Marcin Barylski, Deepak L. Bhatt, Kausik K. Ray, Michael D. Shapiro, Giuseppe Mancia, Gregory Y. H. Lip, Claudio Borghi, Roland E. Schmieder, Krzysztof Narkiewicz, Alina Kuryłowicz

**Affiliations:** 1 Department of Internal Medicine and Clinical Pharmacology, Faculty of Medical Sciences in Katowice, Medical University of Silesia, 40-752 Katowice, Poland; stanislaw.surma@sum.edu.pl; 2 Department of Internal Diseases and Cardiac Rehabilitation, Medical University of Lodz, 90-419 Lodz, Poland; mbarylski3@wp.pl; 3 Mount Sinai Fuster Heart Hospital, Icahn School of Medicine at Mount Sinai, New York, NY 10029, USA; dlbhattmd@post.harvard.edu; 4 Department of Primary Care and Public Health, Imperial Centre for Cardiovascular Disease Prevention, Imperial College, London SW7 2AZ, UK; k.ray@imperial.ac.uk; 5 Department of Cardiovascular Medicine, Wake Forest University School of Medicine, Medical Center Boulevard, Winston Salem, NC 27157, USA; michael.shapiro@wfusm.edu; 6 Department of Medicine and Surgery, University of Milano-Bicocca, 20126 Milan, Italy; giuseppe.mancia@unimib.it; 7 Liverpool Centre for Cardiovascular Science, University of Liverpool, Liverpool L69 3BX, UK; gregory.lip@liverpool.ac.uk; 8 Department of Clinical Medicine, Aalborg University, 9220 Aalborg, Denmark; 9 Department of Cardiology, Lipidology and Internal Medicine with Intensive Coronary Care Unit, Medical University of Bialystok, 15-089 Bialystok, Poland; 10 Department of Medical and Surgical Sciences, University of Bologna, 40126 Bologna, Italy; claudio.borghi@unibo.it; 11 Department of Nephrology and Hypertension, University Hospital Erlangen, Friedrich-Alexander University Erlangen-Nuremberg (FAU), Ulmenweg 18, 91054 Erlangen, Germany; roland.schmieder@uk-erlangen.de; 12 Department of Hypertension and Diabetology, Medical University of Gdańsk, 80-210 Gdańsk, Poland; knark@gumed.edu.pl; 13 Department of Endocrinology, Centre of Postgraduate Medical Education, Ceglowska 8-, 01-813 Warsaw, Poland

**Keywords:** ADIPO-CARE plan, obesity phenotyping, cardiometabolic risk, obesity complications, anti-obesity pharmacotherapy, lifestyle intervention, nutritional therapy, weight management, chronic obesity management

## Abstract

Obesity is a chronic, recurrent, and heterogeneous disease whose effective treatment requires an approach that goes beyond assessment of body mass index and short-term weight loss. Contemporary management should integrate assessment of adipose tissue distribution and function, obesity phenotype, complications, and cardiometabolic risk with individualized nutritional, behavioral, pharmacological, and—if appropriate—surgical interventions. This paper presents the ADIPO–CARE Plan, a practical, structured model for the comprehensive assessment and long-term treatment of people living with obesity. The model comprises two interconnected stages. The ADIPO defines a comprehensive clinical assessment process: A—Assess adiposity, which includes assessing the degree and distribution of excess adipose tissue; D—Define obesity phenotype, which aims to identify the clinical phenotype of obesity; I—Identify complications, which includes detecting obesity-related diseases and complications; P—Profile cardiometabolic risk, which serves to determine global cardiometabolic risk and disease severity; and O—Overall treatment goals, defining individual, realistic therapeutic goals that go beyond weight loss. The second phase, CARE, organizes the treatment and long-term follow-up strategy: C—Comprehensive lifestyle intervention, with particular emphasis on nutritional therapy, physical activity, behavioral interventions, and lifestyle modifications; A—Anti-obesity pharmacotherapy, encompassing the rational use of pharmacotherapy as a complement to lifestyle interventions; R—Response-guided treatment optimization, involving systematic assessment of response and intensification or modification of therapy; and E—Evaluate long-term effectiveness, focusing on maintenance of effects, safety, adherence, relapse prevention, and long-term disease control. The ADIPO–CARE Plan proposes a shift from an approach focused primarily on body weight to a model focused on phenotype, risks, complications, and long-term health benefits. This framework can facilitate the systematization of clinical decisions and the integration of nutritional therapy with other elements of modern, personalized obesity therapy.

## 1. Introduction: Obesity as a Chronic Disease Requiring a New Model of Care

The scale of the global obesity epidemic today underscores the urgent need for new models of disease management. According to data published by the World Health Organisation (WHO), approximately 2.5 billion adults worldwide were affected by overweight in 2022, of whom more than 890 million met criteria for obesity [1]. The prevalence of obesity among adults has more than doubled since 1990. A comparable trend has been observed among children and adolescents aged 5–19 years, in whom the prevalence of excess body weight rose from 8% in 1990 to 20% in 2022, while the prevalence of obesity alone increased from 2% (31 million individuals) to 8% (exceeding 160 million individuals) over the same period [1]. Within the WHO European Region, overweight or obesity now affects 59% of adults and nearly one in three children and is associated with more than 1.2 million deaths annually [2,3]. The economic toll on healthcare systems is similarly considerable: obesity alone was estimated to account for as much as 8% of healthcare expenditure across European Union countries in 2014, and, absent effective intervention, the global costs of overweight and obesity are projected to reach 3 trillion dollars annually by 2030, rising to more than 18 trillion dollars by 2060 [2]. The data presented indicate a persistent discrepancy between progress in obesity treatment and the effectiveness of interventions at the population level. A significant challenge remains the gap between available scientific evidence and its implementation into clinical practice—a gap shaped by barriers at the level of the patient, healthcare professionals, and the healthcare system [4].

**Figure 1:**
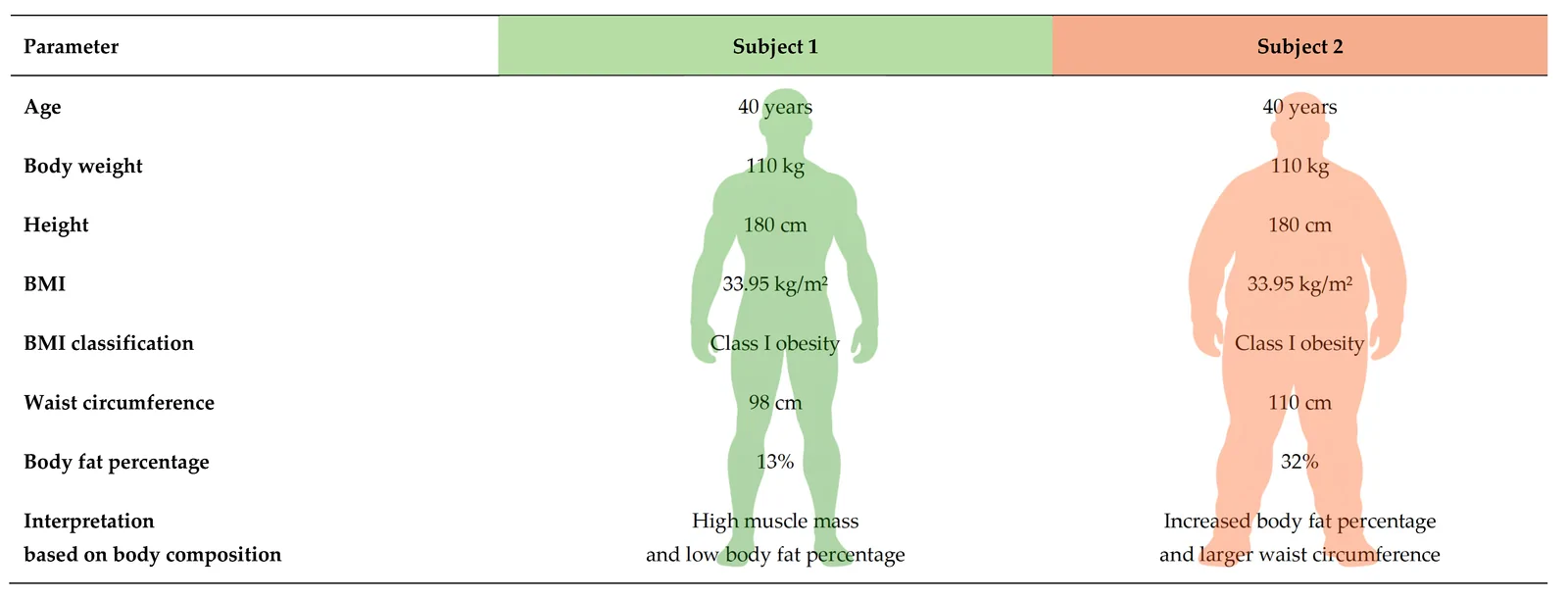
Limitations of body mass index in assessing adiposity and body composition. BMI—body mass index.

Body mass index (BMI) remains a fundamental, easily accessible tool for the screening and classification of obesity, while the percentage of weight loss serves as a key indicator of treatment response [5]. The importance of the latter parameter is underscored by the correlation between the magnitude of sustained weight loss and improvements in metabolic parameters and the clinical course of obesity-related conditions [5]. Nevertheless, BMI does not directly reflect body composition, the distribution of adipose tissue, or the degree of organ dysfunction [6]. Consequently, the modern approach to the diagnosis and treatment of obesity involves supplementing traditional anthropometric measurements and weight-loss targets with an assessment of complications, functional capacity, and individual health risk [6,7]. These limitations may lead to underestimation of adiposity in older adults, individuals with reduced muscle or bone mass, and certain Asian populations, or to overestimation in individuals with high muscle mass (Figure 1) [6].

The limitations of BMI are particularly relevant in specific populations and clinical settings. The relationship between BMI, body fat content, and metabolic risk varies across ethnic groups; for instance, Asian populations may exhibit increased metabolic risk at lower BMI values compared to European populations [6]. BMI can also be an imperfect metric for older adults—where age-related muscle loss may mask excess body fat—and for individuals with significantly high muscle mass, such as athletes, in whom it may lead to an overestimation of obesity [6]. The scale of this issue is well illustrated by a study of 33,896 young athletes, in which obesity was diagnosed in 13.31% of subjects based on BMI, compared to only 5.95% based on body fat percentage (measured via skinfold thickness); notably, 62% of those classified as obese by BMI were false-positive cases [8]. Consequently, the assessment of obesity should not rely solely on BMI but should also consider fat distribution and, where clinically indicated, body composition analysis. This approach is further supported by recent data indicating that measures of central obesity are associated with the presence and progression of subclinical coronary atherosclerosis, as assessed by coronary calcification [9].

These limitations have prompted the emergence of several influential management frameworks over the past decade. Sharma and Kushner proposed the five-stage Edmonton Obesity Staging System (EOSS) in 2009, which evaluates complications, symptoms, psychopathology, functional limitation, and well-being independently of BMI category [10]. Analysis of National Health and Nutrition Examination Survey (NHANES) data involving thousands of adults demonstrated that survival curves diverged more sharply by EOSS stage than by BMI class, with adjusted mortality hazard ratios of 1.57 for stage 2 and 2.69 for stage 3 relative to stage 0/1 [11]. Subsequently, Obesity Canada introduced the 5As of Obesity Management (Ask, Assess, Advise, Agree, Assist), a practical communication and management algorithm designed for primary care [12]. When implemented and tested within a multicentre programme, this approach—following a 90-min training session and the adoption of a supporting toolset—raised the proportion of patients in whom obesity treatment was initiated from 19% to 39% within a single month [13]. Then, in 2016, the American Association of Clinical Endocrinologists and American College of Endocrinology (AACE/ACE) advanced this evolution further with comprehensive guidelines introducing a complications-centric model, in which treatment intensity is calibrated to the presence and severity of reversible complications rather than BMI alone (Complications-Centric Obesity Treatment Algorithm—COTA) [14]. Three years later, the European Association for the Study of Obesity (EASO) popularized the concept of adiposity-based chronic disease (ABCD), proposing a classification framework spanning three dimensions—etiology, degree of adiposity, and health risk—and, in 2024–2026, published updated frameworks for the diagnosis, staging, and management of obesity in adults, complemented by a dedicated pharmacotherapy algorithm [7,15,16,17]. A landmark contribution came with the 2025 Lancet Diabetes & Endocrinology Commission report, which introduced a conceptual distinction between clinical obesity—a chronic systemic disease marked by documented impairment of tissue or organ function—and preclinical obesity, defined as excess adiposity with preserved organ function but heightened risk of future disease [6]. Endorsed by 76 scientific societies and patient organisations, the report nonetheless drew caution from EASO, which warned that labelling certain patients as “preclinical” might delay intervention and restrict access to care [6,18].

Earlier models for managing obesity have been incorporated into clinical recommendations, although the extent of their actual implementation varies. For instance, the EOSS and the 5As framework have been included in Canadian obesity management guidelines [19]. In an implementation study, the use of the aforementioned 5As model led to more frequent initiation of conversations regarding obesity treatment [13]. Nevertheless, a subsequent randomized trial, 5AsT, did not show a significant increase in the frequency of obesity-related consultations (RR = 1.30; 95% CI: 0.83–2.03) [20]. These findings indicate that the formal inclusion of such models in clinical guidelines does not equate to their effective and widespread adoption in clinical practice.

**Figure 2:**
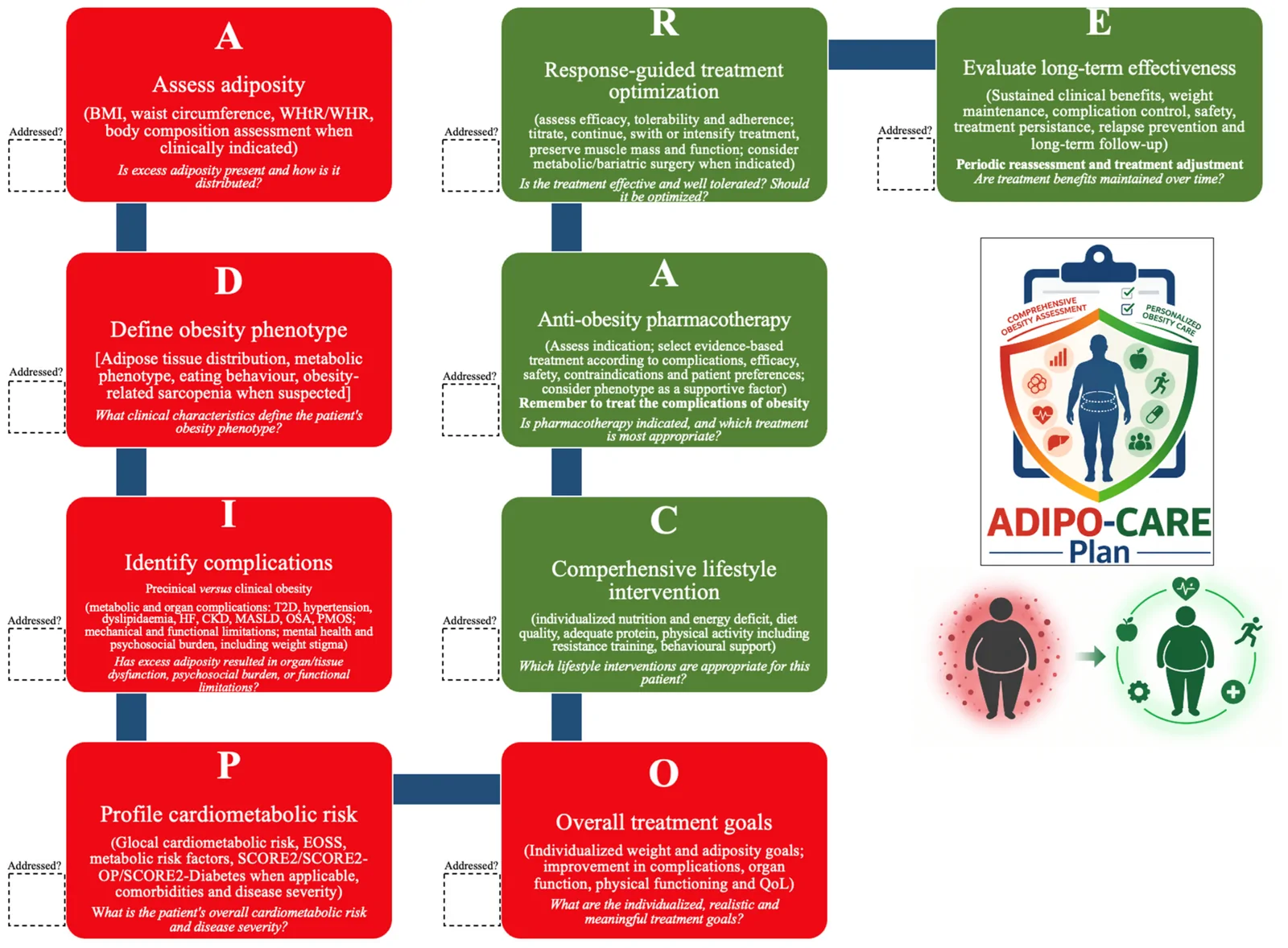
Central illustration. Structured overview of the ADIPO-CARE Plan. BMI—body mass index; CKD—chronic kidney disease; CV—cardiovascular; EOSS—Edmonton Obesity Staging System; HF—heart failure; MASLD—metabolic dysfunction-associated steatotic liver disease; OSA—obstructive sleep apnea; PMOS—polycystic ovary syndrome; QoL—quality of life; SCORE2—Systematic Coronary Risk Evaluation 2; SCORE2-Diabetes, Systematic Coronary Risk Evaluation 2-Diabetes; SCORE2-OP—Systematic Coronary Risk Evaluation 2-Older Persons; T2D—type 2 diabetes; WHR—waist-to-hip ratio; WHtR—waist-to-height ratio.

Existing management models for obesity—including EOSS, 5As, COTA, and ABCD, as well as current EASO recommendations and the proposal from the Lancet Commission—have significantly contributed to the development of a comprehensive, complication-centered approach to the diagnosis and treatment of obesity. While these models differ in their primary purpose and organizational structure, they all share an acknowledgment of the limitations of using BMI as a standalone criterion for assessing the disease, and they emphasize the necessity of considering its metabolic, organ-related, functional, and psychosocial consequences. To address the need for a practical integration of these elements, we propose the ADIPO-CARE Plan (Figure 2/Central Illustration)—a structured, nine-stage clinical management framework consisting of five steps for comprehensive patient assessment (ADIPO) and four steps for the individualization, implementation, and long-term optimization of treatment (CARE). Unlike models that focus primarily on specific aspects—such as assessing disease severity, patient communication, or therapeutic decision-making—the ADIPO-CARE Plan aims to synthesize these complementary components into a cohesive sequence of actions, applicable from the initial consultation through to long-term follow-up. A comparison of the core principles of existing models and the potential added value of the proposed ADIPO-CARE Plan is presented in Table 1. It should be emphasized that this algorithm does not replace current clinical guidelines, nor does it introduce new diagnostic or therapeutic criteria; rather, it serves to organize existing knowledge into a practical framework to support clinical decision-making. The potential value of the ADIPO-CARE Plan lies in integrating the assessment of the severity and characteristics of obesity, the identification of complications, the individualization of treatment, and long-term monitoring into a single, structured clinical process. Such an approach may facilitate the implementation of clinical guidelines while reducing the fragmentation of care and therapeutic inertia. The use of structured management models is supported by findings from implementation research, including studies on the “5As” model [13]. It should be noted, however, that the ADIPO-CARE Plan is a proprietary clinical management model based on current guidelines and medical knowledge. Its feasibility, utility, and potential impact on the quality of care and health outcomes require evaluation in future prospective studies.

### Development of the ADIPO-CARE Plan

The ADIPO-CARE Plan was developed based on a narrative review of the scientific literature, with a particular focus on current international guidelines for the diagnosis and treatment of obesity, existing clinical management models, and studies evaluating the efficacy and safety of therapeutic interventions. Publications were identified primarily through searches in the PubMed database; their selection was guided by their clinical relevance, currency, and utility in constructing the individual stages of the proposed algorithm. The search was narrative in nature and was not conducted according to a systematic review protocol.

Based on an analysis of the gathered literature, an initial version of the nine-stage ADIPO-CARE management pathway was developed, comprising five stages for comprehensive clinical assessment (ADIPO) and four stages for the individualization, management, and long-term optimization of treatment (CARE). The draft algorithm was then shared with invited experts, who were invited to provide comments, suggest changes, or propose additions based on their scientific knowledge and clinical experience. These revisions were incorporated into the final version of the ADIPO-CARE Plan, which was subsequently approved by all participating experts.

The development approach thus combines a narrative synthesis of available scientific evidence with expert evaluation and consensus on the final structure of the proposed management framework. However, this process did not involve a formal consensus methodology, such as the Delphi method, nor did it include a systematic assessment of the quality of evidence or the strength of the recommendations. The ADIPO-CARE Plan should therefore be viewed as an expert-agreed proposal for integrating existing recommendations into a practical clinical management framework, rather than as new guidelines developed through a formal, structured process. Its feasibility, clinical utility, and impact on treatment outcomes require prospective validation.

## 2. Stage I—ADIPO: Comprehensive Clinical Assessment

The practical application of the ADIPO stage should be based on a graded clinical assessment, where the scope of diagnostics depends on the results of the initial evaluation and the patient’s individual circumstances. For all adult patients, the assessment should include a medical history covering the progression of weight gain, comorbidities, current medications, dietary behaviors, physical activity, and functional limitations. It should also involve basic anthropometric measurements—including BMI and an assessment of abdominal obesity—as well as the evaluation of blood pressure and key metabolic parameters. The interpretation of these results must account for age, sex, and ethnic background, particularly regarding adipose tissue distribution and anthropometric thresholds. In cases of ambiguous anthropometric findings, or when an abnormal body composition or sarcopenic obesity is suspected, further assessment of muscle function and body composition—such as bioelectrical impedance analysis (BIA) or dual-energy X-ray absorptiometry (DXA)—should be considered, depending on clinical indications and the availability of these methods. Imaging studies, such as CT or MRI, are not part of routine obesity diagnostics and should be reserved for specific clinical scenarios, including opportunistic use when such images have been acquired for other medical reasons. In-depth functional assessments and specialized phenotyping tests should be reserved for cases where the results are likely to influence the diagnosis, the evaluation of complications, or therapeutic decision-making. This approach facilitates the implementation of the ADIPO-CARE framework in both primary care and specialized clinical settings, without the need to perform every available test on every patient.

### 2.1. A—Assess Adiposity

Rather than resting on BMI classification alone, the model begins by confirming and quantifying excess adipose tissue. The Lancet Diabetes & Endocrinology Commission recommends that, at BMI values at or above conventional obesity thresholds, excess adiposity be confirmed through at least one anthropometric criterion or a direct measurement of body composition [6]. Key complements to BMI include measures of adipose tissue distribution—waist circumference (WC), waist-to-hip ratio (WHR), and waist-to-height ratio (WHtR). A WHO expert consultation on the measurement of waist circumference and waist-to-hip ratio established their predictive value for type 2 diabetes (T2D), cardiovascular disease, and mortality, while accounting for differences by sex, age, and ethnicity [21]. Table 2 presents the corresponding cut-off points for WC and WHR by sex and population group.

The now-familiar clinical message—“waist circumference should be less than half of height”—rests on the generally accepted WHtR threshold of 0.5 in adults and in children from age six; meta-analyses spanning more than 300,000 adults have shown that WHtR identifies cardiometabolic risk more reliably than BMI or waist circumference alone [24]. EASO recommends measuring waist circumference in every individual with a BMI below 35 kg/m^2^ and recognises an elevated risk related to excess adiposity at BMI ≥ 25 kg/m^2^ together with WHtR > 0.5—allowing a diagnosis of obesity even below a BMI of 30 kg/m^2^ when a medical, functional, or psychological complication is present [7].

In situations where anthropometric measurements do not allow for a sufficiently accurate assessment of body composition, the use of additional diagnostic methods may be justified. For instance, in older adults with obesity, reduced muscle strength, or limited functional capacity, one should consider screening for sarcopenic obesity—a process that involves assessing muscle function, followed by an evaluation of body composition using DXA or BIA [25]. These methods can also be useful for monitoring changes in muscle mass during significant weight loss, particularly in patients at risk of sarcopenia [25,26]. Furthermore, CT and MRI scans allow for a detailed assessment of the regional distribution of adipose tissue, including visceral fat; analyzing existing diagnostic images can even provide insights into body composition without the need for additional examinations [27]. Given factors such as cost, limited availability, and—in the case of CT—exposure to radiation, these methods should be applied selectively rather than used for routine screening [6,7]. The clinical relevance of visceral adipose tissue assessment is well illustrated by a CT study at the L3 level involving 1787 individuals, in which visceral obesity (visceral adipose tissue area ≥100 cm^2^) was identified in 45.7% of participants overall—and in more than half of older men and women with a normal BMI—confirming that a normal BMI alone cannot exclude clinically significant excess visceral fat [28].

### 2.2. D—Define Obesity Phenotype

Within the framework of ADIPO-CARE, the characterization of an obesity phenotype should begin with data obtained during a basic clinical assessment, rather than through the routine performance of specialized phenotyping tests. Primary considerations should include the distribution of adipose tissue, metabolic profile, the trajectory of weight gain, eating behaviors, and functional capacity. In older adults—particularly those experiencing reduced muscle strength or limited mobility—the possibility of sarcopenic obesity should also be evaluated. While an in-depth assessment of the mechanisms regulating appetite, satiety, gastric emptying, or energy expenditure may be relevant in certain specialized clinical scenarios, it is not currently a standard or essential component of obesity diagnostics. Identified phenotypic traits should be interpreted in the context of existing complications and overall health risk; they should not be viewed as an independent, validated algorithm for selecting pharmacotherapy.

Obesity is a disease of complex, multifactorial pathogenesis that, according to current concepts, arises from the interaction of genetic and environmental factors, mediated by epigenetic mechanisms. Nevertheless, in a subset of patients it appears possible to identify the mechanism that plays the decisive role in their weight gain. Identifying the dominant mechanisms underlying weight gain could support the individualization of obesity treatment; however, its utility in predicting the response to specific anti-obesity medications requires further validation.

A study conducted at the Mayo Clinic and published in 2021 attempted to characterise obesity phenotypes by the primary driver of weight gain and to compare the efficacy of phenotype-guided pharmacotherapy with treatment selected according to standard guidelines [29]. The study was carried out in two stages: first, 450 patients with obesity underwent testing to determine their disease phenotype, after which the efficacy of phenotype-based treatment was compared with standard care. To identify the individual phenotypes, the investigators employed: (i) an assessment of satiation, based on the number of calories consumed until the sensation of maximal fullness and cessation of eating during an unrestricted buffet-style test meal; (ii) analysis of gastric emptying of solids (as a determinant of postprandial satiety and the time to hunger recurrence); (iii) an evaluation of hedonic eating behaviour using the Hospital Anxiety and Depression Scale (HADS) and the Three-Factor Eating Questionnaire (TFEQ-21), together with validated behavioural questionnaires assessing emotional eating; and (iv) measurement of resting energy expenditure by indirect calorimetry.

**Figure 3:**
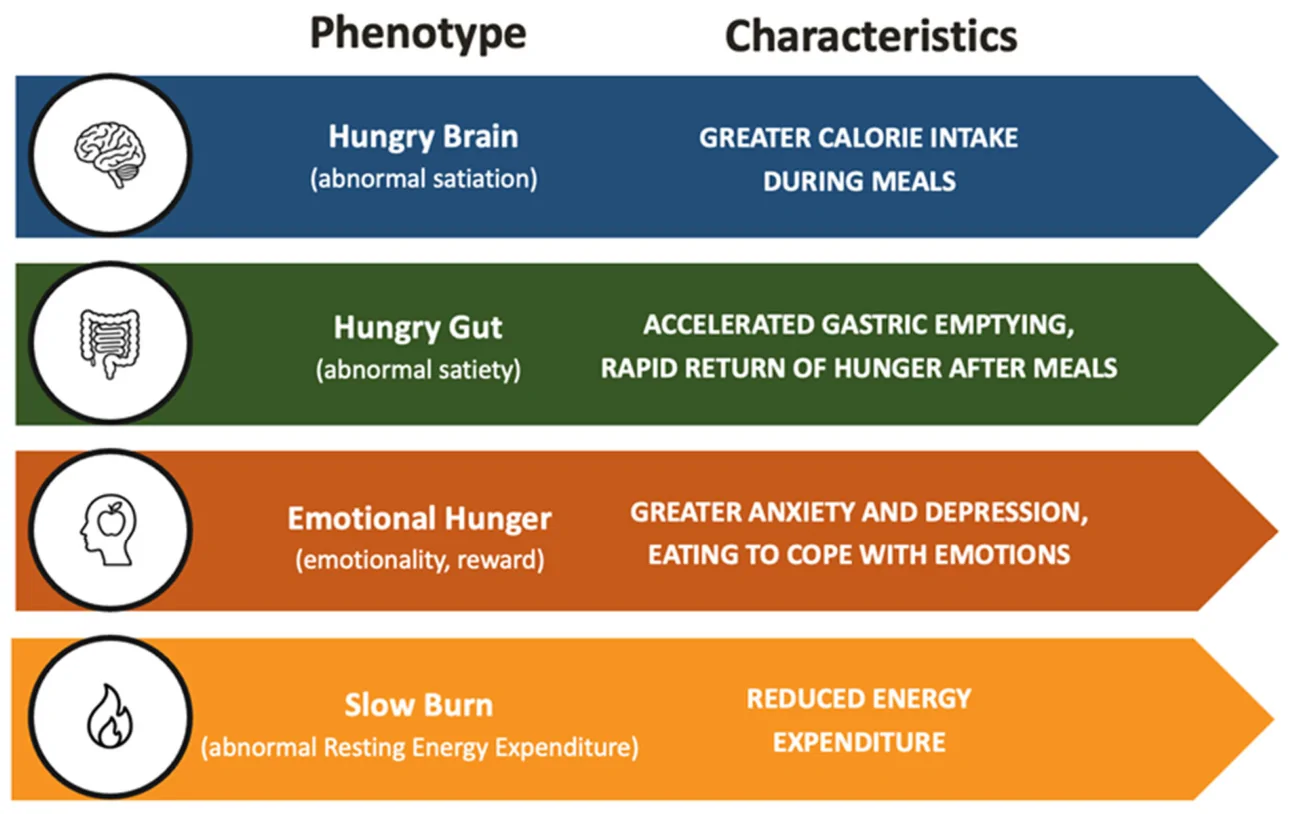
Obesity phenotypes defined by the primary driver of weight gain [29].

Based on these assessments, participants were assigned to one of four phenotypes (Figure 3).

The prevalence of the individual phenotypes within the study population was broadly similar, ranging from 12% to 18%. A mixed phenotype was identified in 27% of participants, while in 15% no dominant phenotype could be established [29].

In the second stage of the study, 312 patients were compared—those receiving treatment tailored to their obesity phenotype (*n* = 84) and those receiving standard care (*n* = 228). This comparison was non-randomized, and there were differences between the groups in certain baseline characteristics [29]. Patients with the “hungry brain” phenotype, who accounted for 40% of the personalised-treatment group, were offered the centrally acting agent lorcaserin at a dose of 20 mg, or phentermine–topiramate at a dose of 7.5 + 46 mg/day. Patients with “emotional hunger” (30% of participants) received bupropion–naltrexone (180 + 16 mg twice daily). For the “hungry gut” phenotype (18% of the group), a glucagon-like peptide-1 receptor agonist (GLP-1 RA)—liraglutide, titrated to a target dose of 3 mg—was proposed, one of whose mechanisms of action is to slow gastric emptying. Patients with a reduced metabolic rate (12% of participants) received phentermine at 15 mg/day. For patients with a mixed phenotype, the investigators selected pharmacotherapy based on the dominant phenotype. After 12 months of treatment, phenotype-guided treatment selection was associated with 1.75-fold greater weight loss than non-phenotype-guided care (mean weight reduction of 15.9% vs. 9.0%) [29]. These findings are preliminary and should be interpreted with caution, given the non-randomized study design, the limited size of the phenotype-based treatment group, and the potential influence of confounding factors [29]. Furthermore, the response to obesity pharmacotherapy varies significantly between individuals, and the utility of phenotyping for predicting the efficacy of specific medications has not yet been sufficiently validated [29]. Therefore, in the ADIPO-CARE Plan, phenotyping is proposed as a tool to support the individualization of treatment, rather than as a mandatory algorithm for selecting pharmacotherapy.

As part of a comprehensive characterization of the obesity phenotype, current pharmacotherapy must also be systematically analyzed, as certain medications can promote weight gain or limit the effectiveness of weight-loss efforts. Key examples include selected antipsychotics—particularly olanzapine and clozapine—antidepressants (including tricyclic antidepressants and mirtazapine), certain antiepileptic drugs (such as pregabalin, gabapentin, and valproates), systemic glucocorticosteroids, selected beta-blockers, and certain hypoglycemic agents, including insulin, sulfonylureas, and thiazolidinediones [30]. The clinical significance of this issue is highlighted by data from the “All of Us” program, showing that 36% of overweight or obese individuals were taking at least one medication associated with weight gain, while approximately 20% were taking more than one such drug [31]. Furthermore, in the Look AHEAD study, exposure to at least one weight-gain-promoting medication was associated with a lower likelihood of achieving clinically significant weight loss [32].

Phenotyping, however, extends beyond metabolic drivers of weight gain to include patterns of adipose tissue distribution. Visceral adiposity is more strongly associated with insulin resistance and cardiometabolic disease than peripheral or subcutaneous fat accumulation, whereas subcutaneous expansion with minimal inflammatory infiltration and limited visceral involvement characterises an insulin-sensitive pattern known as “metabolically healthy” obesity. Adipocyte hypertrophy, inflammation, fibrosis, restricted expansion capacity, and ectopic lipid deposition together define adipose tissue dysfunction—a dimension of disease severity that BMI alone cannot capture [33].

It is also worth noting body weight phenotypes based on the coexistence of metabolic disorders. These include metabolically healthy normal weight (MHNW), metabolically unhealthy normal weight (MUNW), metabolically healthy overweight/obesity (MHO), and metabolically unhealthy overweight/obesity (MUO) (Table 3) [34].

The prevalence of metabolically healthy obesity (MHO) and metabolically unhealthy obesity (MUO) phenotypes varies significantly across studies, depending on the definition of metabolic health adopted, the number of permissible metabolic abnormalities, and the characteristics of the study population, including age and sex. Although some studies have estimated that approximately 10–20% of individuals with obesity fall into the MHO category, these figures should not be considered universal, nor should they be used as diagnostic thresholds. The classification of MHO/MUO should be based on an individual assessment of metabolic parameters, while taking into account the limitations and heterogeneity of the definitions employed [35]. It is important not to equate the metabolic phenotyping of obesity with the classification into preclinical and clinical obesity recently proposed by the Lancet Commission. The categories of MHO and MUO primarily refer to the metabolic profile of an individual with obesity. Although there is no single universally accepted definition of MHO/MUO in the literature, these classifications primarily take into account abnormalities such as elevated blood pressure, impaired glucose metabolism, hypertriglyceridemia, and low HDL-C levels, with some definitions also including insulin resistance or markers of inflammation [34,35]. In contrast, the Lancet Commission classification is not intended primarily to characterize the metabolic profile or stratify future risk, but rather to determine whether excess adiposity has already led to clinically manifest disease [6]. Clinical obesity is defined as a state in which excess adiposity is accompanied by objective signs or symptoms of tissue or organ dysfunction resulting from obesity and/or significant, age-adjusted impairment of daily functioning [6]. This means that the two systems describe different, albeit partially overlapping, dimensions of obesity. An individual meeting the criteria for MHO may still have clinical obesity if, despite a relatively normal metabolic profile, they exhibit dysfunction—such as respiratory or musculoskeletal impairment—associated with excess adiposity. Conversely, the mere presence of a single metabolic abnormality or risk factor does not automatically equate to a diagnosis of clinical obesity unless clinically significant organ or functional dysfunction associated with obesity can be demonstrated [6]. Therefore, MHO/MUO and preclinical/clinical obesity should be viewed as complementary rather than competing approaches: the former characterizes the patient’s metabolic phenotype, while the latter determines whether excess adiposity has already translated into clinically manifest disease [6,34,35,36]. In the ADIPO-CARE Plan framework, this distinction is reflected in the sequence of steps: stage D—Define obesity phenotype—involves characterizing the phenotype (including the metabolic phenotype), whereas the subsequent stage I—Identify complications—entails a systematic search for obesity-related organ and functional dysfunctions, thereby enabling the distinction between preclinical and clinical obesity.

Moreover, in older patients or those presenting additional risk factors, sarcopenic obesity warrants deliberate and specific evaluation. The ESPEN/EASO consensus defines this entity as the coexistence of excess adipose tissue with diminished muscle mass and/or function, recommending an initial assessment of muscle function followed by formal body-composition analysis—with DXA as the preferred method, BIA as a second-line alternative, and opportunistic use of previously acquired CT imaging where available [25].

### 2.3. I—Identify Complications

**Figure 4:**
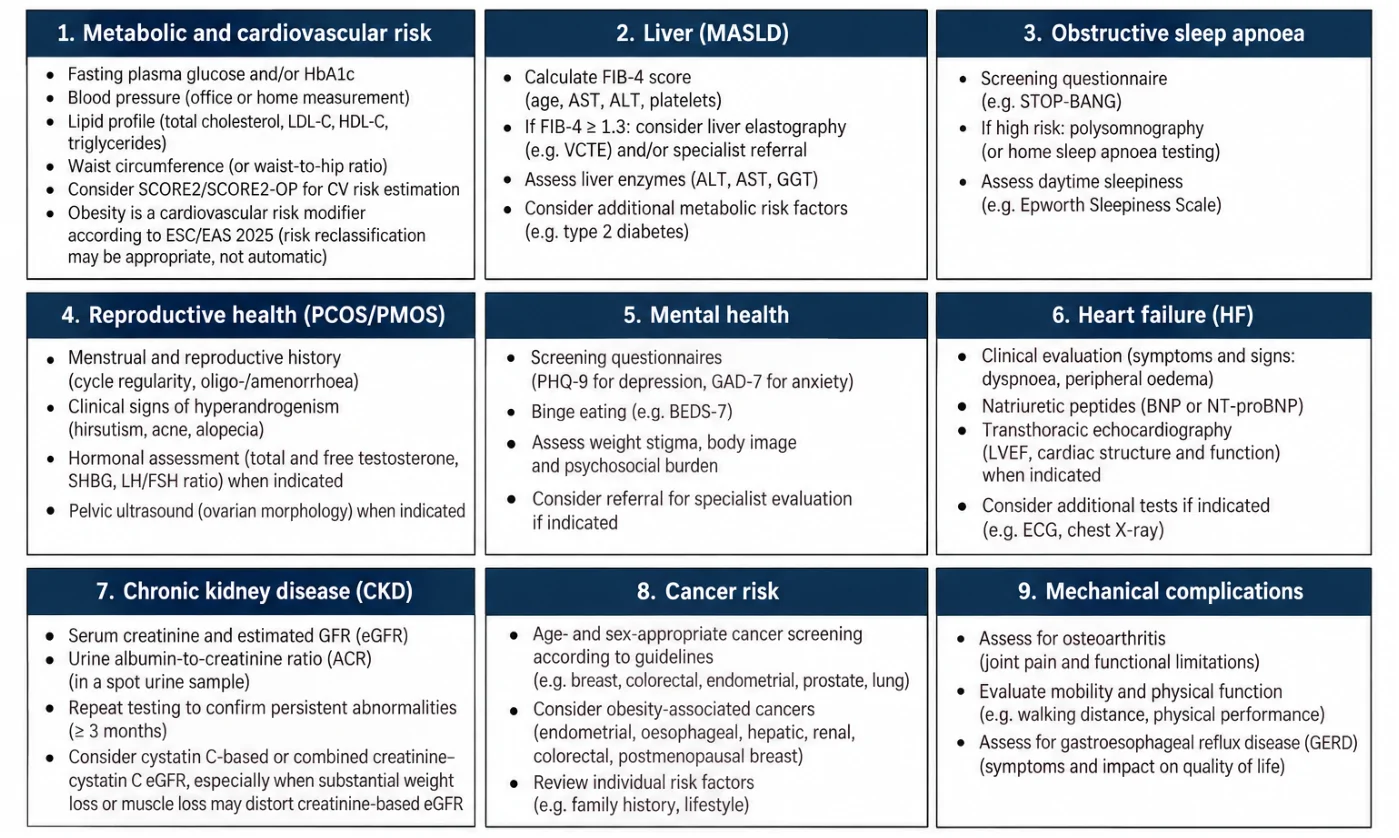
Basic screening for obesity-related complications. ACR—albumin-to-creatinine ratio; ALT—alanine aminotransferase; AST—aspartate aminotransferase; BEDS-7—Binge Eating Disorder Screener-7; BNP—B-type natriuretic peptide; CKD—chronic kidney disease; CKD-EPI—Chronic Kidney Disease Epidemiology Collaboration; ECG—electrocardiogram; eGFR—estimated glomerular filtration rate; FIB-4—Fibrosis-4 Index; FSH—follicle-stimulating hormone; GAD-7—Generalized Anxiety Disorder-7; GGT—gamma-glutamyl transferase; HbA1c—glycated haemoglobin; HDL-C—high-density lipoprotein cholesterol; HF—heart failure; LDL-C—low-density lipoprotein cholesterol; LH—luteinizing hormone; LVEF—left ventricular ejection fraction; MASLD—metabolic dysfunction-associated steatotic liver disease; NT-proBNP—*N*-terminal pro-B-type natriuretic peptide; PCOS—polycystic ovary syndrome; PHQ-9—Patient Health Questionnaire-9; PMOS—polyendocrine metabolic ovarian syndrome; SHBG—sex hormone-binding globulin; STOP-BANG—Snoring, Tiredness, Observed apnoea, high blood Pressure, Body mass index, Age, Neck circumference, and Gender; TC—total cholesterol; VCTE—vibration-controlled transient elastography.

The third ADIPO component involves systematically identifying obesity-related complications (Figure 4). Metabolic assessment should include glycaemia/HbA1c, blood pressure, and a fasting lipid profile, since the Lancet Commission criteria for clinical obesity incorporate a metabolic cluster comprising hyperglycaemia, elevated triglycerides, and low HDL cholesterol [6]. The American Heart Association (AHA) likewise identifies obesity as a direct determinant of dyslipidaemia, T2D, and hypertension, and highlights visceral adiposity in particular as an independent marker of poorer cardiovascular prognosis [37].

Given the strong pathophysiological and epidemiological link between excess adiposity and heart failure (HF), evaluation for HF represents an important component of the assessment of obesity-related cardiovascular complications. Obesity is a major risk factor for HF, with a particularly strong association with the cardiometabolic phenotype of heart failure with preserved ejection fraction (HFpEF) [38,39]. The diagnosis may be challenging because symptoms and signs frequently encountered in people with obesity—including exertional dyspnoea, fatigue, exercise intolerance, and peripheral oedema—may overlap with those of HF [39]. In patients with symptoms or signs suggestive of HF, current ESC guidelines recommend measurement of natriuretic peptides and transthoracic echocardiography as key components of the diagnostic work-up [38]. Importantly, obesity is associated with lower circulating concentrations of BNP and NT-proBNP; therefore, natriuretic peptide levels should be interpreted with particular caution in this population, and low values may not reliably exclude HF, especially HFpEF [38,39]. Consequently, unexplained dyspnoea, reduced exercise capacity, signs of congestion, or other features suggestive of HF in a person living with obesity should prompt further cardiovascular evaluation rather than being attributed solely to excess body weight.

With respect to the liver, the EASL–EASD–EASO guidelines define MASLD (metabolic dysfunction-associated steatotic liver disease) as steatotic liver disease occurring in the presence of at least one cardiometabolic risk factor and in the absence of harmful alcohol consumption [40]. Active case-finding is recommended in patients with type 2 diabetes and in those with obesity accompanied by additional metabolic risk factors, beginning with blood-based fibrosis scores such as FIB-4, followed by transient elastography to confirm or exclude advanced fibrosis.

Obesity is a significant, independent risk factor for the development and progression of chronic kidney disease (CKD). In a meta-analysis involving over 630,000 individuals with initially normal kidney function, obesity was associated with a 28% increased risk of a GFR decline to <60 mL/min/1.73 m^2^ and a 51% increased risk of albuminuria; furthermore, each 1 kg/m^2^ increase in BMI raised the risk of CKD by approximately 2% [41]. Importantly, the risk depends on the metabolic phenotype: “metabolically healthy” obesity increased the risk of CKD by approximately 30%, whereas obesity accompanied by metabolic abnormalities increased it by 63% [41]. Results from a 20-year follow-up study confirm the particularly adverse impact of the chronic coexistence of obesity and metabolic abnormalities [42]. The pathophysiology of kidney injury in obesity primarily involves glomerular hyperfiltration, linked to factors such as activation of the sympathetic nervous system and the renin-angiotensin-aldosterone system, as well as increased sodium reabsorption [43]. Chronic hyperfiltration leads to glomerulomegaly, podocyte injury, and glomerulosclerosis. Clinically, obesity-related glomerulopathy (ORG) manifests primarily as proteinuria—sometimes reaching nephrotic-range levels, though usually without the full clinical picture of nephrotic syndrome—and is characterized by glomerulomegaly and frequently focal segmental glomerulosclerosis (FSGS) [43]. Assessment of renal function in an individual with obesity should take into account both eGFR and albuminuria/proteinuria (albumin-to-creatinine ratio, ACR), as eGFR may be normal or elevated due to hyperfiltration in the early stages. When interpreting renal risk, it is also important to consider the limitations of BMI—which does not reflect visceral fat distribution or muscle mass and strength—as this can lead to an underestimation of risk, particularly in cases of sarcopenic obesity [43].

A history focused on obstructive sleep apnoea (OSA)—covering snoring, witnessed apnoeas, and daytime sleepiness—together with referral for polysomnography when indicated, is likewise integral to the assessment. The American Academy of Sleep Medicine reports that a 10% weight gain is associated with a sixfold increase in OSA risk, and that undiagnosed OSA can lead to systemic hypertension while raising the risk of stroke, heart failure, atrial fibrillation, and coronary artery disease. Screening should therefore not be omitted on the basis of a low BMI alone, particularly in people of Asian ancestry or those presenting suggestive symptoms [44].

Furthermore, the evaluation of metabolic complications should take into account symptoms of osteoarthritis, limitations in mobility, and complaints suggestive of gastroesophageal reflux disease, with further diagnostic steps determined by the clinical presentation [6,19].

Assessment of complications should further extend to menstrual and ovulatory disorders and polyendocrine metabolic ovarian syndrome (PMOS). PMOS (previously defined as polycystic ovarian syndrome—PCOS) had been recognised as an established chronic complication at EOSS stage 2, and the 2023 international PCOS guidelines underscore its metabolic, cardiovascular, sleep-apnoea-related, and psychological dimensions [45]. The recognition of PCOS as a cardiometabolic disease is reflected in its new designation, established in 2026: polyendocrine metabolic ovarian syndrome (PMOS) [46].

Mental health assessment stands as an integral pillar of comprehensive obesity evaluation, reflecting the well-established bidirectional relationship between excess adiposity and psychiatric morbidity. A landmark meta-analysis of 15 longitudinal studies (58,745 participants) demonstrated that obesity increases the risk of incident depression by 55%, whereas depression, in turn, independently raises the risk of subsequent obesity by 58%—confirming a reciprocal, self-reinforcing pathway between the two conditions [47]. In light of this evidence, the Canadian Adult Obesity Clinical Practice Guidelines recommend routine screening for depression, anxiety, binge eating disorder, attention-deficit/hyperactivity disorder, and trauma history in all individuals presenting for obesity management, recognising that undetected psychiatric comorbidity may quietly undermine treatment adherence and outcomes [48]. Validated instruments such as the Patient Health Questionnaire (PHQ-9) and Generalized Anxiety Disorder-7 (GAD-7) lend themselves readily to efficient first-line screening for depressive and anxiety symptoms in routine practice, while brief eating-disorder screens—the BEDS-7 (Binge Eating Disorder Screener-7) for instance—should be employed to identify binge eating disorder, a condition that disproportionately affects patients seeking obesity treatment and warrants a therapeutic approach distinct from standard behavioural weight management. In addition to identifying mental health disorders, it is also important to consider the psychosocial consequences of obesity—specifically weight-related stigma, experiences of discrimination, and low self-esteem. In appropriate cases, the brief, three-item Weight Bias Internalization Scale (WBIS-3) can be a useful tool for assessing internalized weight stigma [49]. While this instrument complements the assessment of mental health, it does not replace a clinical interview or the formal diagnosis of mental health disorders. For individuals being considered for bariatric surgery, a structured pre-surgical mental health evaluation by a qualified clinician is recommended to identify risk factors for poor postoperative outcomes, with monitoring continued thereafter for psychiatric symptoms, eating psychopathology, substance use disorders, and suicidal ideation [48].

Finally, cancer assessment should include age- and sex-appropriate screening, along with a review of individual risk factors. The US National Cancer Institute notes that greater adipose tissue mass is associated with at least 13 cancer types; marked obesity, in particular, increases the risk of type 1 endometrial cancer up to sevenfold and of oesophageal cancer nearly fivefold relative to individuals of normal body weight [50]. Accordingly, EASO recommends regular cancer screening in people living with obesity [7].

Figure 4 illustrates the basic methods for assessing obesity-related complications.

It is worth emphasizing that an accurate assessment of the presence of obesity-related complications (clinical obesity) is crucial. Recent data provide empirical justification for this approach. An analysis incorporating NHANES 2017–2018 data and the EPIC-Potsdam cohort revealed that over 80% of individuals with confirmed obesity met the criteria for clinical obesity. In the prospective EPIC-Potsdam analysis, clinical obesity was associated with an approximately 2.8-fold higher risk of cardiovascular disease and a nearly 7.9-fold higher risk of type 2 diabetes compared to individuals without obesity who did not meet clinical criteria. Notably, individuals with pre-clinical obesity did not exhibit an increased risk of cardiovascular disease, although the risk of T2D remained significantly elevated [51]. These findings further indicate that merely confirming excess adiposity is insufficient for a complete clinical assessment and should be complemented by a systematic search for its organ-related consequences.

### 2.4. P—Profile Cardiometabolic Risk

The fourth component of the ADIPO-CARE plan integrates the preceding data into an assessment of overall risk and disease severity. Cardiometabolic risk stratification lies at the heart of obesity assessment, for BMI alone offers a poor proxy for the metabolic consequences of excess—and, more pointedly, ectopic—adiposity.

The need for such an integrated assessment is further reinforced by the contemporary concept of the cardiovascular–kidney–metabolic (CKM) syndrome. The 2026 AHA/ACC/ADA/ASN guidelines define CKM as an interconnected continuum encompassing metabolic risk factors—including obesity and T2D—CKD, and cardiovascular disease [52]. This approach emphasizes that individual disorders should not be evaluated merely as independent comorbidities, given their pathophysiological and prognostic interconnections. From this perspective, the “P” stage of the ADIPO-CARE Plan should integrate cardiovascular risk assessment with the evaluation of metabolic status and renal function, thereby enabling a more comprehensive risk characterization and the identification of individuals requiring particularly intensive, multifaceted management [52].

The American Heart Association’s scientific statement on obesity and cardiovascular disease underscores that it is visceral and ectopic fat deposition, rather than total adiposity per se, that drives insulin resistance, dyslipidaemia, and hypertension, and accordingly recommends waist circumference as a routine adjunct to BMI in everyday clinical practice [37].

In keeping with this view, the Lancet Commission’s diagnostic criteria for clinical obesity identify a distinct metabolic cluster—hyperglycaemia, elevated triglycerides, and depressed HDL cholesterol—together with elevated blood pressure, as tangible evidence of organ dysfunction attributable to excess adiposity [6].

The harmonised definition of metabolic syndrome, which requires at least three of five criteria—elevated waist circumference, triglycerides ≥150 mg/dL, HDL cholesterol below 40 mg/dL in men or 50 mg/dL in women, blood pressure ≥130/85 mmHg, and fasting glucose ≥100 mg/dL—provides a practical, readily applicable framework for identifying patients at markedly heightened cardiovascular and diabetes risk [53].

EOSS remains a five-stage (0–4) clinical severity scale that integrates cardiometabolic and mechanical complications with functional limitations, well-being, and psychopathology, rather than treating BMI class as the primary determinant of disease severity [11]. Stage 0 indicates no identifiable risk factors or functional limitations; stage 1—subclinical risk factors (e.g., prediabetes, metabolic syndrome, MASLD, or borderline hypertension) and mild limitations; stage 2—established chronic diseases such as type 2 diabetes, hypertension, sleep apnea, PMOS, or osteoarthritis; and stages 3 and 4 indicate, respectively, organ damage/substantial disability and severe, potentially terminal disability [54].

In individuals without established atherosclerotic cardiovascular disease (ASCVD) or diabetes, current European guidelines recommend SCORE2 to estimate the 10-year risk of fatal and non-fatal cardiovascular events in adults aged 40–69 years, while SCORE2-OP is used in those aged 70–89 years [55,56,57]. In patients with T2D aged 40–69 years without established ASCVD or severe target-organ damage, cardiovascular risk should instead be estimated using the dedicated SCORE2-Diabetes algorithm, which integrates conventional cardiovascular risk factors with diabetes-specific variables, including age at diabetes diagnosis, HbA1c, and estimated glomerular filtration rate (eGFR) [58,59]. Importantly, neither SCORE2 nor SCORE2-Diabetes has been developed or validated for individuals younger than 40 years, and these algorithms should not be extrapolated to this age group. Because 10-year absolute cardiovascular risk in young adults is strongly influenced by age and may therefore underestimate cumulative lifetime cardiovascular burden, risk assessment in this population should focus on the presence and severity of major risk factors and risk modifiers, with consideration of lifetime cardiovascular risk [55]. Accordingly, in patients with T2D aged <40 years without ASCVD or severe target-organ damage, current ESC guidance recommends an individualized assessment of ASCVD risk factors rather than application of SCORE2-Diabetes [58].

Importantly, the 2025 ESC/EAS Focused Update on dyslipidaemia management explicitly identifies obesity as a cardiovascular risk modifier beyond SCORE2/SCORE2-OP estimation. Its presence may support upward reclassification of cardiovascular risk, particularly in individuals at moderate risk or close to treatment decision thresholds, potentially influencing LDL-C treatment goals and the intensity of lipid-lowering therapy [57]. Thus, identifying obesity has implications not only for weight management but also for cardiovascular risk stratification and preventive treatment decisions.

In summary, a comprehensive evaluation should combine anthropometric measurement with metabolic parameters, ideally complemented by validated global cardiovascular risk calculators and functional assessment, to calibrate the intensity of subsequent metabolic and lifestyle intervention.

### 2.5. O—Overall Treatment Goals

The final component of the ADIPO framework translates clinical assessment into individually tailored therapeutic goals. The Lancet Diabetes & Endocrinology Commission holds that treatment goals for clinical obesity should be individualised, aimed at improving clinical manifestations, achieving remission wherever possible, and forestalling disease progression or organ damage [6]. Therapeutic success is thus judged by objective improvement or remission of clinical manifestations, rather than by weight loss alone or by surrogate risk markers.

Remission is defined as partial or complete resolution of the clinical and laboratory evidence of obesity-related tissue or organ dysfunction. It is not, however, synonymous with cure, and the degree of weight reduction required to achieve substantial improvement or remission has not been unequivocally established, likely varying across cardiovascular, metabolic, and musculoskeletal manifestations [6].

In preclinical obesity, the principal aim is risk reduction rather than correction of established disease, with treatment intensity calibrated to the degree and distribution of adiposity, age, ethnicity, family predisposition, coexisting obesity-related disorders, and a careful weighing of benefit against risk [6].

European guidelines for the management of obesity in adults continue to regard a 5–10% weight reduction as a clinically meaningful initial target, yet explicitly caution against a narrow focus on weight or BMI alone. Goals should instead encompass waist circumference, fat mass and fat-free mass, comorbidities, quality of life, and overall well-being, alongside a realistic, individualised long-term plan to sustain benefit and prevent relapse [7,15,16,17]. Table 4 summarises selected goals in obesity treatment.

In practice, the “O” component of the ADIPO model should articulate a jointly agreed set of goals tailored to phenotype and disease severity—for instance, control or remission of diabetes, improvement in blood pressure or lipid profile, reduction of fibrosis risk in MASLD, attenuation of sleep-apnoea severity, improved pain and mobility, restoration of capacity for activities of daily living, and enhanced mental well-being and quality of life—together with a clearly specified target change in body weight or adiposity calibrated to support these outcomes, rather than treating a fixed percentage of weight loss as the sole endpoint.

## 3. Stage II—CARE: Treatment Strategy and Long-Term Follow-Up

An accurate diagnosis is the essential first step toward proper obesity treatment, and it can be achieved through a comprehensive clinical assessment guided by the ADIPO algorithm. Based on this approach, we developed the CARE protocol, which focuses on patients’ treatment strategy and long-term follow-up.

### 3.1. C—Comprehensive Lifestyle Intervention

Comprehensive lifestyle intervention (CLI) constitutes the cornerstone of obesity treatment: this evidence-based model integrates a dietary plan incorporating an energy deficit, increased physical activity, and behavioural therapy as complementary components of a unified strategy, rather than treating any one element in isolation [61]. Accordingly, the recommended model of care for patients with obesity is collaborative, delivered within a multidisciplinary team in which physicians, dietitians, physiotherapists, and psychologists or psychodietitians jointly contribute to the treatment process [7,14,15]. Such collaboration may take various forms: American guidelines, for instance, recommend at least six months of engagement with a qualified specialist, encompassing a minimum of 14 individual or group sessions during the weight-loss phase, followed by monthly or more frequent contact for at least a further year during the maintenance phase—though the frequency and duration of visits are often tailored to the individual patient’s needs [62].

Within this framework, dietary counselling should aim to establish an energy deficit within a balanced dietary pattern, typically in the range of 500–750 kcal/day, corresponding to intakes of 1200–1500 kcal/day for women and 1500–1800 kcal/day for men, with higher targets within these ranges reserved for individuals with greater body weight or physical activity levels [7,14,15]. However, the recommended energy deficit should be viewed as an indicative starting point rather than a universal therapeutic goal. Its magnitude should be determined on an individual basis, taking into account baseline energy requirements, age, sex, physical activity level, comorbidities, metabolic complications, and the risk of malnutrition and sarcopenia. Evaluating the patient’s current pharmacotherapy is of particular importance. Medications that affect body weight, appetite, or glucose metabolism can alter both the need for nutritional intervention and its safety profile. In patients receiving insulin or sulfonylurea derivatives, restricting energy and carbohydrate intake may necessitate adjustments to antidiabetic treatment to mitigate the risk of hypoglycemia. Conversely, when using incretin-based therapies—such as GLP-1 receptor agonists or dual GIP/GLP-1 receptor agonists—reduced appetite, early satiety, and gastrointestinal side effects may lead to an inadequate intake of protein, fluids, and micronutrients. In such cases, further increasing the energy deficit should not be an automatic objective; instead, the priority shifts toward ensuring the quality and nutritional value of the diet, treatment tolerability, and the preservation of muscle mass and function. The intensity of the intervention should be periodically adjusted based on the clinical response, the rate of weight loss, nutritional status, and the patient’s individual capacity [63,64]. Very low-calorie diets (VLCDs), providing fewer than 800 kcal per day and typically administered as meal replacements, are not routinely recommended in contemporary obesity treatment protocols, except in select cases involving preparation for bariatric surgery. Although VLCDs can produce striking short-term results—weight losses of up to 20 kg within 12 weeks—they confer no guaranteed advantage over moderate caloric-deficit diets in the long term, and carry an increased risk of nutritional deficiencies [7,14,15]. Diet composition has also evolved through clinical debate. Lifestyle modification programs have traditionally favoured low-fat, high-carbohydrate diets [65]; however, recent research has shown that a range of alternative approaches—including low-carbohydrate/high-protein, Mediterranean-style, Dietary Approaches to Stop Hypertension (DASH), low-glycemic-load, and portion-controlled diets—may be equally effective, provided each achieves an energy deficit. Indeed, when caloric intake is matched, macronutrient composition itself confers little additional benefit for weight loss [7,14,15]. Macronutrient composition may nonetheless influence improvements in cardiometabolic risk factors, so dietary selection should consider both an individual’s cardiometabolic profile and personal preferences. The importance of a flexible approach to selecting a dietary model is supported by the findings of the randomized DIETFITS trial, which included 609 adults with excess body weight. After 12 months, no significant difference in weight loss was observed between a healthy low-fat diet and a healthy low-carbohydrate diet (−5.3 kg and −6.0 kg, respectively). Notably, the genetic variants analyzed and baseline insulin secretion did not predict greater efficacy for any specific dietary model [66]. While these results do not rule out the benefits of personalizing a diet, they suggest that such personalization should be based primarily on clinical needs, nutritional quality, patient preferences, cultural factors, economic accessibility of food products, and the feasibility of long-term adherence—rather than on unvalidated methods for predicting individual responses. Further evidence is provided by a three-year analysis of the randomized PREDIMED-Plus trial, involving 1521 participants; in this study, an intervention combining a calorie-reduced Mediterranean diet, increased physical activity, and behavioral support resulted in a greater reduction in body fat percentage (a between-group difference of −0.38 percentage points) and visceral fat mass (−70.4 g) compared to the control approach [67]. These findings indicate that the effectiveness of lifestyle interventions should also be evaluated in terms of changes in body composition, rather than solely by total weight loss.

In terms of macronutrients, a key aspect of nutrition, particularly during pharmacological treatment for obesity, is ensuring adequate protein intake to prevent lean body mass loss. According to the 2025 Consensus of the European Association for the Study of Obesity (EASO), the recommended protein intake during such treatment should be between 1.2 and 1.5 g per kg of adjusted body weight [16]. A practical way of controlling macronutrients in the diet involves interventions that use visual plate models to help control portion sizes. A meta-analysis of randomised trials found that teaching patients to control the composition of their meals and portion sizes using ‘dietary plates’ leads to sustained benefits in terms of weight loss, glucose and lipid levels, and finally—cardiovascular effects [68].

Overall, dietary interventions should be individualized according to clinical characteristics, nutritional requirements, patient preferences, and the feasibility of long-term adherence. Beyond weight reduction, their effectiveness should be assessed in terms of cardiometabolic health, body composition, and preservation of muscle function [69,70].

Physical activity is another essential component of CLI, complementing rather than replacing dietary modification. While physical activity alone yields only modest weight loss in the absence of caloric restriction, it remains important for cardiometabolic health and for sustaining weight loss over time: at least 150 min *per* week of moderate-to-vigorous activity (e.g., walking, cycling or swimming) is recommended during the weight-loss phase, rising to 200–300 min per week during the maintenance phase [7,14,15]. In addition to these general guidelines, it is important to bear in mind that patients suffering from obesity often have limited mobility, so the targets set must not be too ambitious. It is therefore sensible to first assess the patient’s baseline level of physical activity and then adapt the training programme to their capabilities. Increasing physical activity levels—even by simply raising the daily step count (by 500 steps/day)—is also associated with significant cardiovascular benefits [71]. Moreover, as part of the prevention of sarcopenia during pharmacological treatment for obesity, resistance training is also recommended, ideally two to three times a week. This should comprise one to three sets of resistance exercises, with eight to 12 repetitions in each set [72]. Elderly people, patients with limited physical fitness and people at risk of sarcopenia require special individualization, for whom the benefits of reducing body fat should be balanced with the risk of loss of muscle mass and function. A meta-analysis of 25 randomized clinical trials showed that adding resistance training to a dietary intervention limited the loss of lean body mass (SMD 0.40), increased the reduction of fat mass (SMD −0.36), and improved muscle strength, despite no significant additional effect on overall body weight reduction [73]. These results emphasize that the intensity of energy restriction and the type and load of physical activity should be adjusted to age, initial fitness, comorbidities and exercise tolerance. Especially during pharmacotherapy leading to significant weight loss, it is important to monitor not only body weight, but also the adequacy of protein intake, nutritional status and muscle function, with appropriate modification of the intervention in the event of deterioration of these parameters [63,64].

Behavioural therapy should likewise be deliberately structured rather than assumed as a default component of care. During the weight-loss phase, key techniques include collaborative, specific goal setting; daily monitoring of caloric intake and physical activity; at least weekly weight monitoring; feedback; problem-solving; stimulus control; and cognitive techniques to address distorted thinking. In the maintenance phase, emphasis shifts toward continued monitoring, problem-solving, cognitive restructuring, and relapse prevention, with motivational interviewing and acceptance-based approaches offering additional support for engagement and adherence [7,14,15].

The evidence for comprehensive lifestyle intervention (CLI) in obesity treatment is not merely suggestive but compelling, anchored in randomised trials and systematic reviews that together establish clinically meaningful and durable benefit. A pooled analysis conducted for the U.S. Preventive Services Task Force, synthesising 67 trials and more than 22,000 participants, found that behaviour-based interventions produced significantly greater weight loss at 12–18 months than usual care (mean difference −2.39 kg; 95% CI −2.86 to −1.93), while also attenuating weight regain during subsequent maintenance (mean difference −1.59 kg; 95% CI −2.38 to −0.79) [74]. This body of evidence underlies the Task Force’s recommendation that clinicians offer or refer adults with obesity to intensive, multicomponent behavioural programmes. Yet the value of CLI reaches well beyond the scale. In the Diabetes Prevention Program, an intensive lifestyle intervention combining dietary modification, physical activity, and behavioural support reduced the incidence of T2D by 58% among high-risk adults over a mean follow-up of 2.8 years—a margin that notably exceeded the 31% reduction conferred by metformin over the same period [75]. Remarkably, this protective effect proved durable rather than transient: at 15 years, the Diabetes Prevention Program Outcomes Study found diabetes incidence among those originally assigned to lifestyle intervention still 27% lower than among placebo-arm participants [76]. Weight loss itself appears to be the principal mediator of this benefit, with each kilogram lost associated with an approximate 16% reduction in diabetes risk, independent of concurrent dietary and activity changes [77]. It is worth noting that the 9-month lifestyle intervention (Tübingen Lifestyle Intervention Programme) reduced the proportion of individuals with clinical obesity from 71% to 57% and the proportion of those with prediabetes from 52% to 29% [36].

Together, these findings recast CLI not as a mere strategy for weight management, but as a durable, disease-modifying intervention—one whose sustained benefits justify its continued centrality within any comprehensive framework for obesity care.

### 3.2. A—Anti-Obesity Pharmacotherapy

**Figure 5:**
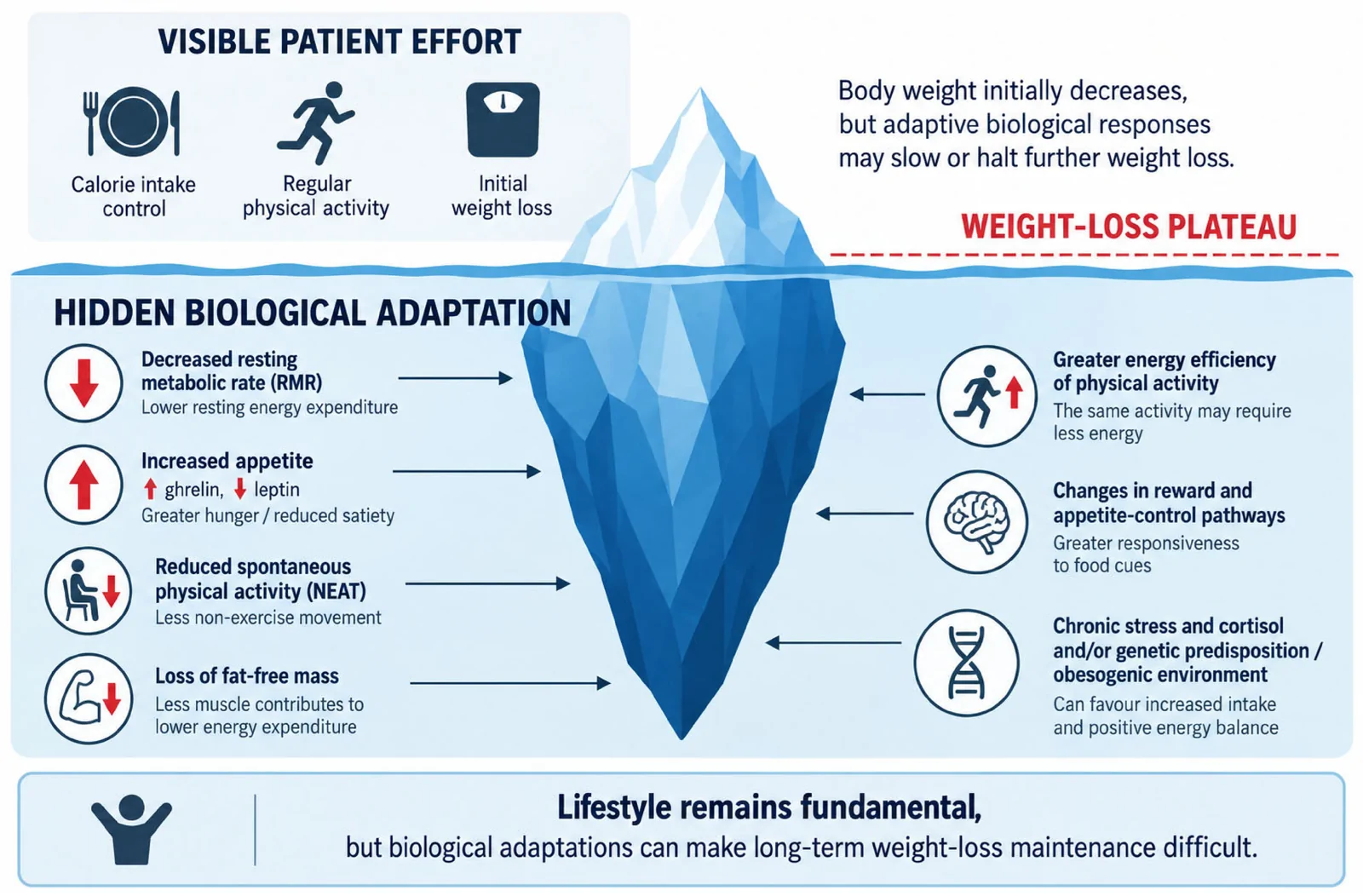
Biological adaptations opposing long-term weight-loss maintenance. Based on information from [78,79].

Given the neuroendocrine compensatory mechanisms that evolved specifically to guard against energy loss, lifestyle interventions alone are frequently insufficient to secure sustained weight loss over the long term (Figure 5) [78,79]. It is worth recalling, moreover, that the principal aim in treating patients with obesity is not weight loss per se, but rather the optimisation of the cardiometabolic profile and, by extension, the prevention of chronic complications. For many patients, therefore, pharmacotherapy constitutes an indispensable component of obesity management.

Pharmacotherapy is indicated as an adjunct to comprehensive lifestyle intervention in adults with a BMI of ≥30 kg/m^2^, or ≥27 kg/m^2^ in the presence of at least one weight-related comorbidity—such as type 2 diabetes, hypertension, dyslipidaemia, obstructive sleep apnoea (OSA), or metabolic dysfunction-associated steatotic liver disease (MASLD). These thresholds are harmonised across the principal international guidelines [7,15,16,17], as well as in the recently published joint statement of The Obesity Society, the Obesity Medicine Association, and the Obesity Action Coalition [80]. Notably, certain population-specific guidelines—including those issued for East Asian cohorts—recommend lower BMI cut-offs (≥25 kg/m^2^, or ≥23 kg/m^2^ with a comorbidity), recognising the elevated metabolic risk observed at comparatively lower adiposity in these populations [81]. Beyond BMI itself, eligibility for pharmacological treatment also requires documented failure to achieve clinically meaningful weight loss through lifestyle intervention alone, the absence of contraindications to the agent under consideration, and realistic patient commitment to sustained, long-term therapy.

It must be emphasized that anti-obesity pharmacotherapy should be part of an integrated management strategy and cannot replace targeted treatment—aligned with current guidelines—of obesity-related complications and coexisting cardiometabolic risk factors. Disorders and diseases identified during the comprehensive ADIPO assessment—specifically T2D, hypertension, dyslipidemia, chronic kidney disease, MASLD/MASH, OSA, and overt cardiovascular disease—should be treated concurrently, in accordance with current recommendations specific to each condition. This approach allows weight loss to be viewed not as an isolated therapeutic goal, but as one element of a comprehensive strategy aimed at improving metabolic health, limiting organ damage, and reducing overall cardiometabolic risk.

The choice of anti-obesity pharmacotherapy should extend beyond a comparison of expected weight loss among the various drugs. According to the current EASO approach, the therapeutic decision should focus primarily on the patient’s individual clinical profile and the complications associated with excess body weight—stemming from both mechanical load and adipose tissue dysfunction [16]. This approach has been further elaborated in the EASO framework for the pharmacological management of obesity (updated in 2026), which is based on 62 randomized clinical trials. In this model, the choice of therapy takes into account not only expected weight loss but also available evidence regarding the impact of specific drugs on particular obesity-related complications, including cardiovascular disease, HFpEF, obstructive sleep apnea, knee osteoarthritis, and MASLD/MASH [17]. At the same time, the therapeutic decision should consider safety, contraindications, treatment availability, and the patient’s individual preferences.

In those at high cardiovascular risk, the agent of choice is the glucagon-like peptide-1 receptor agonist (GLP-1RA) semaglutide—a conclusion established by the multicentre SELECT trial, published in 2023, which found that weekly subcutaneous injections of 2.4 mg semaglutide in overweight or obese patients with documented cardiovascular disease, but without diabetes, reduced the risk of major cardiovascular events by 20% [5]. Notably, this benefit proved independent of weight loss itself, pointing to a pleiotropic mechanism plausibly mediated through the attenuation of inflammation. Subsequent studies have further demonstrated a favourable effect of semaglutide on quality of life among patients with obesity and coexisting heart failure [82], while tirzepatide, the dual GLP-1/GIP (glucose-dependent insulinotropic peptide) receptor agonist, has likewise been shown to reduce both the frequency of heart failure exacerbations and mortality from this cause [83]. Further significant data regarding the cardiovascular effects of tirzepatide were provided by the SURPASS-CVOT trial, which included 13,165 patients with T2D and established atherosclerotic cardiovascular disease. Tirzepatide was non-inferior to dulaglutide—a GLP-1 receptor agonist with documented cardiovascular benefits—regarding the risk of MACE-3 (cardiovascular death, myocardial infarction, or stroke): events occurred in 12.2% and 13.1% of patients, respectively (HR = 0.92; 95.3% CI: 0.83–1.01; *p* = 0.003 for non-inferiority), without demonstrating a statistically significant superiority of tirzepatide over dulaglutide (*p* = 0.09) [84]. However, a prespecified indirect analysis using data from SURPASS-CVOT and REWIND estimated that, compared with imputed placebo, tirzepatide was associated with a 28% lower risk of MACE-3 (HR = 0.72; 95% CI: 0.55–0.94), as well as a lower risk of a composite endpoint comprising cardiovascular death or heart failure events (HR = 0.70; 95% CI: 0.51–0.96) and all-cause mortality (HR = 0.61; 95% CI: 0.45–0.82) [85]. However, given the indirect nature of the comparison with an imputed placebo, these results should be interpreted as complementary to the primary randomized comparison of tirzepatide versus dulaglutide, rather than as evidence derived from a direct tirzepatide-versus-placebo study. Semaglutide and tirzepatide have proved effective in halting the progression of MASLD [86,87], as well as the progression from pre-diabetes to overt diabetes [88,89]—an effect also documented for liraglutide, a GLP-1RA administered via daily injection [90]. Liraglutide and tirzepatide have similarly demonstrated efficacy in the treatment of OSA [91,92], while semaglutide is further supported by clinical trial evidence for its role in the management of knee osteoarthritis [93] and symptomatic peripheral artery disease [94]. An increasing body of evidence also points to the potential renal benefits of incretin-based therapies. In a prespecified analysis of the SELECT trial—which included individuals with overweight or obesity and established cardiovascular disease, but without diabetes—semaglutide 2.4 mg reduced the risk of a composite renal endpoint by 22% compared with placebo [95]. Furthermore, in a randomized trial involving individuals with overweight or obesity and CKD (without diabetes), 24 weeks of treatment with semaglutide reduced albuminuria by 52.1% relative to placebo [96]. These findings suggest that potential renal benefits may serve as an additional factor to consider when tailoring pharmacotherapy for obesity, particularly in patients with comorbid CKD.

Alongside this complications-guided algorithm—which essentially represents a “damage control” strategy—an alternative, phenotype-guided approach has been proposed, generally reserved for individuals without evident complications. This strategy was first tested by Acosta and colleagues in a nonrandomised trial of 312 patients, phenotyped according to the principal pathophysiological mechanism underlying their weight gain into four subgroups—“hungry brain”, emotional hunger, “hungry gut”, and “slow burn”—and demonstrated that phenotype-guided pharmacotherapy yielded a mean weight loss of 15.9% at 12 months, compared with 9.0% in the absence of phenotype guidance [29]. It is worth noting that in the study by Acosta et al., phentermine monotherapy (15 mg/day) was also used in patients with a “slow burn” phenotype (characterized by reduced energy expenditure). However, its use in clinical practice is limited by national regulatory status and drug availability [29].

This finding lends support to individualising drug selection according to eating behaviour, satiation and satiety, metabolic rate, comorbidities, contraindications, and patient preference, although the single-centre, pragmatic design of the trial, together with the specific drug combination employed, warrants caution before regarding these phenotypes as universally validated criteria for treatment selection.

In patients for whom emotional eating is a dominant phenotypic feature, the use of naltrexone combined with bupropion may be considered, taking into account the indications, contraindications, and the individual clinical profile. It should be emphasized, however, that the emotional eating phenotype alone does not constitute a validated criterion for selecting this pharmacotherapy. Owing, however, to its noradrenergic component—and the attendant risk of elevated heart rate and blood pressure—this combination is contraindicated in patients with uncontrolled hypertension or cardiac instability [97]. In individuals with reduced resting energy expenditure, phentermine/topiramate has been proposed as a potential therapeutic option within phenotype-guided treatment strategies. However, evidence supporting the selection of this combination specifically on the basis of this phenotype remains insufficient, and treatment decisions should primarily reflect established indications, contraindications, and the patient’s overall clinical profile [98].

Despite significant progress in anti-obesity pharmacotherapy and the availability of highly effective treatment methods, global epidemiological projections continue to point toward an increase in the number of people living with obesity. According to analyses from the Global Burden of Disease study, the number of adults aged 25 and older living with obesity could reach approximately 1.95 billion by 2050 [99]. At the same time, recent observations from the United States suggest a possible reversal of the prevailing epidemiological trend. According to a report from the Gallup National Health and Well-Being Index, the prevalence of obesity among American adults declined from 39.9% in 2022 to 37.0% in 2025—a reduction corresponding to an estimated 7.5 million fewer people living with obesity. Concurrently, the proportion of adults reporting the use of GLP-1 receptor agonist medications for weight loss increased from 5.8% in early 2024 to 12.4% in 2025 [100]. While the convergence of these trends may suggest a potential population-level impact of modern anti-obesity pharmacotherapy, the observational nature of the data precludes the confirmation of a causal relationship. The discrepancy between the persistent global rise in the burden of obesity and these initial, favorable epidemiological signals in specific populations underscores the importance of access to effective therapies, appropriate patient selection, and long-term continuity of treatment. In this context, the ADIPO-CARE Plan offers a structured clinical approach aimed at individualizing and optimizing therapy; its actual impact on treatment outcomes, however, requires prospective validation in the future.

Table 5 summarises the evidence from clinical trials underpinning the selection of specific agents in the pharmacological treatment of obesity.

### 3.3. R—Response-Guided Treatment Optimization

Effective monitoring of obesity pharmacotherapy demands a structured, dose-titration-aligned follow-up schedule paired with clearly defined criteria for treatment continuation or discontinuation. During dose escalation, patients should be reviewed about every four weeks to assess gastrointestinal tolerability, blood pressure, and heart rate, since these adverse effects—rather than weight loss itself—typically limit the rate of early treatment progress [101]. Treatment efficacy should be formally reassessed once the maximum tolerated or maintenance dose has been reached, generally at three months: European guidelines define an adequate early response as a weight loss of at least 5% of baseline body weight in non-diabetic patients, or at least 3% in those with diabetes, with therapy continued in responders and discontinued in those who fail to meet this threshold [101,102]. This principle has since been carried forward into the current EASO framework for the pharmacological treatment of obesity, which situates ongoing response assessment within a broader, complications-oriented staging of the disease and emphasises individualised therapeutic targets over anthropometric criteria alone [7,16]. Patients who fail to reach the response threshold should either be up-titrated to the next available dose or switched to an alternative agent, whereas early responders should continue therapy long-term, reflecting the understanding of obesity pharmacotherapy as chronic disease management rather than a time-limited intervention [101]. Beyond weight itself, ongoing monitoring should encompass waist circumference—a practical surrogate for visceral adiposity and an independent marker of cardiometabolic risk—alongside blood pressure, glycaemic and lipid parameters, hepatic enzymes, and kidney function, with particular attention in patients experiencing gastrointestinal losses [102].

The growing number of available therapies further underscores the importance of systematically assessing treatment response and individualizing subsequent management. A systematic review and network meta-analysis published in 2026—covering 262 randomized clinical trials, 99,791 participants, and 19 drugs—demonstrated significant differences among therapies regarding weight loss, other clinical outcomes, and tolerability [103]. These findings highlight that the response to pharmacotherapy should not be evaluated solely based on the percentage change in body weight. Decisions regarding whether to continue, increase the dose, switch, or intensify treatment should take into account the overall clinical response, including improvements in obesity-related complications, treatment tolerability and safety, effects on body composition, and the patient’s individual goals and preferences.

Assessment of treatment response should also account for potential changes in the reliability of kidney function estimates during substantial weight loss. Reductions in skeletal muscle mass may decrease creatinine generation and serum creatinine concentrations, leading to an apparent improvement in creatinine-based estimated glomerular filtration rate (eGFR) without a corresponding increase in measured GFR [104]. Accordingly, changes in creatinine-based eGFR should be interpreted alongside body composition and other markers of kidney health, including albuminuria. In patients with substantial weight loss or suspected changes in muscle mass, cystatin C-based or combined creatinine–cystatin C estimates may provide complementary information, although these markers also have limitations [104,105].

It should also be noted that, despite the impressive results observed in clinical trials, individual responses in real-world practice vary considerably. In controlled trials of high-dose semaglutide or tirzepatide, approximately 10% of participants fail to achieve even a 5% total body weight loss [106,107], while non-response appears to be even more frequent in real-world cohorts, where lower adherence, more conservative dosing, and a greater burden of comorbidities converge to attenuate treatment effect. A weight-response framework has accordingly been proposed, stratifying patients into four categories: non-responders (<5% total body weight loss (TBWL) at 16–24 weeks on the maximum tolerated dose), modest responders (5–10% TBWL), good responders (10–20% TBWL), and super-responders (>20% TBWL, occasionally exceeding 30%). Independent of this final outcome, patients may also be distinguished as early or late responders—the former achieving clinically meaningful metabolic and weight targets within the first 12 weeks, the latter falling short initially yet frequently attaining success with continued treatment [108]. Recognising these distinct trajectories is essential to optimising titration schedules and preventing premature discontinuation of therapy. Indeed, early weight-loss trajectories—particularly the magnitude of loss at weeks 4, 8, and 16—carry considerable predictive weight for long-term outcomes: data from the STEP trial programme indicate that patients who fail to achieve at least 5% weight loss by week 16 rarely reach clinically meaningful endpoints by week 68, marking this juncture as a clinically appropriate decision point for therapeutic modification [106]. Factors that determine response to obesity pharmacotherapy are complex, encompassing baseline metabolic phenotype, sex (women generally respond more favourably to treatment), hormonal status, comorbidities and concomitant treatments, microbiome composition, and genetic predisposition [109].

One of the significant factors limiting the effectiveness of pharmacotherapy for obesity is poor tolerability. The most common adverse effects associated with GLP-1 receptor agonists and tirzepatide are gastrointestinal symptoms, primarily nausea, constipation, and diarrhea. These are typically mild and tend to occur during dose escalation; however, in some patients, they may persist, hindering the achievement of a therapeutic dose [110]. Management strategies include gradual dose escalation, dietary modifications, and treatment tailored to the specific type and severity of the symptoms. In cases of persistent nausea or vomiting—despite these interventions—short-term symptomatic treatment (such as the use of an antiemetic or prokinetic agent) may be considered, following an individual assessment of indications, contraindications, and safety. It should be noted, however, that evidence regarding the efficacy of prokinetics for managing adverse effects of incretin-based therapies is limited, and their routine use is not recommended [110]. If symptoms persist, it may be appropriate to delay further dose increases, reduce the dose, or temporarily discontinue treatment. The risk of biliary complications also remains an important safety consideration. A meta-analysis of 76 randomized clinical trials involving 103,371 participants revealed an increased risk of gallbladder and biliary tract disease associated with the use of GLP-1 receptor agonists (RR = 1.37; 95% CI: 1.23–1.52), particularly in trials focused on weight loss (RR = 2.29; 95% CI: 1.64–3.18) [111]. These observations complement findings from an analysis of the FAERS database by Tao et al., which identified a signal for the disproportionately frequent reporting of cholelithiasis and cholecystitis, though a causal link could not be confirmed [112]. Although the UPGRADE trial evaluated the prophylactic use of ursodeoxycholic acid (UDCA) following bariatric surgery, it did not demonstrate a statistically significant reduction in the risk of symptomatic cholelithiasis across the entire study population (6.5% vs. 9.7%; RR = 0.67; 95% CI: 0.43–1.04) [113]. These results should not be directly extrapolated to the pharmacotherapy of obesity, as there is currently insufficient evidence to justify routine UDCA prophylaxis during incretin-based treatment. From a cardiological perspective, the potential for an increase in resting heart rate during the use of GLP-1 receptor agonists and tirzepatide should also be considered, warranting an individualized clinical assessment—especially in patients with advanced cardiovascular disease [114]. The most critical concern associated with pharmacotherapy for obesity, however, remains the risk of sarcopenia arising from excessively rapid weight reduction. Weight loss encompasses not only adipose tissue but, to a meaningful degree, fat-free mass as well, which may result in diminished muscle strength and reduced physical function. Patients over 65 years of age face the greatest risk of sarcopenia, particularly those with a history of prolonged immobilization or chronic conditions associated with heightened catabolism, such as CKD. In these populations, non-pharmacological measures are particularly important—namely, education on adequate protein intake and implementation of a safe resistance-training programme. Substantial loss of muscle mass during obesity treatment may also, paradoxically, impair myocardial exercise reserve [72].

Nonetheless, randomized and observational studies in recent years have not confirmed that pharmacotherapy for obesity with GLP-1RAs and tirzepatide is associated with an increased risk of acute pancreatitis or malignancy [115]. At the same time, the increasingly widespread use of incretin-based therapies has given rise to new safety signals requiring further monitoring. In the case of semaglutide, particular attention has been drawn to non-arteritic anterior ischemic optic neuropathy (NAION), which the European Medicines Agency has classified as a very rare adverse reaction, although the absolute risk remains low [116,117]. Meanwhile, the potential link between GLP-1 receptor agonists and thyroid cancer—including medullary thyroid carcinoma—remains inconclusive: current data from randomized clinical trials do not confirm a significant increase in risk; however, given the rarity of these events and the limited duration of follow-up, a potential long-term risk cannot be definitively ruled out [118,119]. These rare safety signals underscore the importance of long-term pharmacovigilance as the use of incretin-based therapies continues to expand.

### 3.4. E—Evaluate Long-Term Effectiveness

Long-term evaluation of obesity treatment should encompass not only anthropometric parameters but also clinical endpoints (defining complications related to excess body mass), adverse events, adherence and treatment persistence, as well as functional and patient-relevant outcomes. As described earlier, modern pharmacotherapy for obesity enables not only a substantial—often exceeding 20%—reduction in body weight, but also remission of many obesity-related complications: resolution of carbohydrate metabolism disorders, improvement in lipid profile, control of blood pressure and OSA symptoms, and a reduction in the severity of MASLD [7,15,16,17,59]. This raises a central question: once the treatment goal for obesity has been achieved, should pharmacotherapy be continued?

Available data unequivocally indicate that pharmacologically induced weight loss should not be regarded as a permanent effect that persists after the cessation of therapy. A systematic review and meta-analysis published in 2026—covering 37 studies, 63 intervention arms, and 9341 participants—observed weight regain averaging approximately 0.4 kg per month following the discontinuation of obesity medications; with newer, more effective incretin-based therapies, this rate reached approximately 0.8 kg per month [120]. Notably, weight regain following the end of pharmacotherapy occurred more rapidly than after behavioral weight-loss interventions, and was accompanied by the gradual loss of some of the cardiometabolic benefits achieved [116]. Furthermore, an independent meta-analysis and meta-regression published in 2026 demonstrated that, within one year of discontinuing GLP-1 receptor agonists, patients regained on average approximately 60% of the weight lost during treatment [121]. These findings further confirm the chronic and relapsing nature of obesity and indicate that effective weight loss should mark the beginning of a long-term maintenance and relapse-prevention phase, rather than the conclusion of therapy.

Treatment-withdrawal studies confirm that anti-obesity pharmacotherapy should be regarded, for many patients, as chronic treatment rather than a finite course. In the STEP 1 extension phase, participants who discontinued semaglutide 2.4 mg after 68 weeks regained 11.6% of body weight over the following year off treatment—approximately two-thirds of their prior weight loss—with cardiometabolic improvements partially reversing in parallel [122]. The recently published SURMOUNT-MAINTAIN study extended this line of inquiry, comparing three post-treatment scenarios in patients who had successfully reduced body mass after one year on the maximum tolerated dose of tirzepatide [119]. Participants switched from tirzepatide to placebo after 12 months regained weight steadily over the following year; 67% of this group regained at least half of their previously lost weight, necessitating “rescue” treatment to prevent a full relapse into obesity. By contrast, those who remained on their maximum tolerated dose (10 or 15 mg) retained virtually all of their prior weight loss over the subsequent year, ultimately losing an average of approximately 22% of baseline weight, with only 8% requiring intensified treatment due to significant regain. Patients whose dose was reduced to 5 mg also preserved the majority of the treatment effect relative to placebo—averaging a 17% reduction from baseline after the additional year, meaningfully better than the roughly 10% seen with placebo—though this effect proved less durable than full-dose continuation: the 5 mg group regained, on average, 5.6 kg more than those maintained on the full dose, and the proportion requiring rescue therapy was three times higher (25% versus 8%). In other words, reducing the dose of an anti-obesity medication is not a neutral maintenance strategy: it is associated with somewhat weaker weight control and a substantially greater likelihood of needing additional treatment. This yields a key strategic conclusion from SURMOUNT-MAINTAIN: dose de-escalation should not be treated as a standard or “natural” step once a target is reached, but rather as an individualised solution for patients for whom sustaining a higher dose is difficult—whether owing to adverse effects, personal preference, or constraints on medication availability or cost [123].

A further therapeutic option under evaluation in the maintenance phase of obesity treatment is orforglipron, a once-daily, oral, non-peptide small-molecule GLP-1 receptor agonist. The phase 3b ATTAIN-MAINTAIN trial assessed orforglipron as a strategy for preserving weight loss previously achieved with injectable semaglutide or tirzepatide in patients who had reached a weight plateau. At 52 weeks, participants who transitioned to orforglipron retained substantially more of their prior weight loss than those switched to placebo: those coming from semaglutide maintained a model-based estimate of 74.7% of their original weight reduction, compared with only 49.2% among placebo recipients, while those transitioning from tirzepatide maintained 79.3% versus 37.6% with placebo [124]. These findings position orforglipron as a promising oral maintenance option capable of consolidating the benefits achieved with injectable incretin-based therapies, offering a pharmacological bridge for long-term weight management without the burden of continued subcutaneous administration—an attribute of particular relevance given the chronic, relapsing nature of obesity and the well-documented risk of weight regain upon treatment discontinuation.

Notwithstanding the rapid evolution of pharmacotherapy, bariatric/metabolic surgery (MBS) remains an important and durable benchmark, as well as a treatment-intensification option, in the management of obesity. Joint guidelines from the American Society for Metabolic and Bariatric Surgery and the International Federation for the Surgery of Obesity and Metabolic Disorders (ASMBS/IFSO) recommend MBS for individuals with a BMI ≥ 35 kg/m^2^, irrespective of the presence, absence, or severity of obesity-related comorbidities, and advise its consideration in class I obesity (BMI 30–34.9 kg/m^2^) when metabolic disease is present or when non-surgical treatment has failed to achieve substantial or durable weight loss and comorbidity improvement [125].

The durable metabolic benefits of surgical treatment are well illustrated by the randomized STAMPEDE trial, which compared intensive medical therapy alone with medical therapy supplemented by Roux-en-Y gastric bypass (RYGB) or sleeve gastrectomy in patients with obesity and T2D. After 5 years, the target HbA1c level of ≤6.0% was achieved by 29% of patients who underwent RYGB, 23% of those who underwent sleeve gastrectomy, and only 5% of patients treated with medical therapy alone [126]. Furthermore, surgical treatment was associated with greater and more sustained weight loss and a reduced need for antidiabetic medications. However, the benefits of metabolic surgery extend beyond glycemic control. In the randomized GATEWAY trial involving patients with obesity and hypertension, 73% of patients who underwent RYGB achieved at least a 30% reduction in the number of antihypertensive medications while maintaining blood pressure control after 3 years, compared with 11% of those treated with medication alone (RR = 6.52; 95% CI: 2.50–17.03; *p* < 0.001). Hypertension remission—defined as blood pressure <130/80 mmHg without pharmacotherapy—was achieved in 31% and 0% of patients, respectively [127]. Importantly, the benefits of metabolic surgery are also reflected in long-term hard clinical endpoints. In the prospective Swedish Obese Subjects (SOS) study, surgical treatment was associated with a lower risk of cardiovascular mortality (HR = 0.47; 95% CI: 0.29–0.76) and of a first fatal or non-fatal myocardial infarction or stroke (HR = 0.67; 95% CI: 0.54–0.83) versus standard care [128]. During a median follow-up of approximately 24 years, bariatric surgery was associated with lower all-cause mortality (HR = 0.77; 95% CI: 0.68–0.87) and cardiovascular mortality (HR = 0.70; 95% CI: 0.57–0.85), and the adjusted median life expectancy was 3.0 years longer than in the group receiving conservative treatment [129]. These results underscore that, despite the unprecedented development of effective anti-obesity pharmacotherapy, metabolic surgery remains a vital, evidence-based therapeutic option for carefully selected patients, enabling not only sustained weight loss but also the improvement or remission of significant metabolic complications of obesity and reducing the risk of major cardiovascular events.

Eligibility for surgical treatment should therefore remain an integral part of the long-term care pathway for suitable patients, rather than being presented solely as a final option after repeated unsuccessful courses of pharmacotherapy.

A notable advance brought about by modern pharmacotherapy is the ability to address weight regain following gastric bypass surgery at an early stage, substantially reducing the number of revision procedures required. This reflects the current approach to long-term evaluation of obesity treatment, which favours sustained lifestyle intervention (and pharmacotherapy when appropriate) together with proactive self-monitoring, intervening before weight regain becomes established.

## 4. The Place of the ADIPO-CARE Model in Clinical Practice

Despite an increasingly better understanding of the pathophysiology of obesity, its recognition as a chronic, relapsing disease, and the unprecedented development of effective treatments, a significant gap remains between available knowledge and guidelines and their actual implementation in clinical practice. The American Heart Association has identified this issue as a major “implementation gap” occurring at multiple levels—patient, physician, healthcare system, and social environment—emphasizing that advancements in obesity science and therapeutic options have not yet fully translated into consistent application in daily practice [4]. The problem begins at the stage of diagnosis and the initiation of discussions regarding treatment. In the UK cohort of the ACTION-IO study, only 47% of individuals with obesity had discussed their weight with a healthcare professional in the preceding five years. Of these, 49% had previously received a formal diagnosis of obesity, representing just 24% of the total study population. Furthermore, a follow-up visit was scheduled for only 19% of those who had such a discussion, even though 62% desired such a visit and 96% stated they would have attended one had it been scheduled [130].

Recent data indicate that this gap persists even in the era of modern pharmacotherapy. In the ACTION-Ireland study, individuals with obesity waited an average of eight years from the onset of initial weight-related concerns to their first discussion with a healthcare professional. While physical activity and dietary interventions were recommended by 72% and 67% of healthcare professionals, respectively, only 34% recommended anti-obesity pharmacotherapy, 23% suggested a consultation with an obesity specialist, and 24% recommended bariatric treatment. Among the individuals with obesity themselves, only 10% used prescription medications, 4% consulted a specialist, and 2–4% underwent bariatric surgery [131]. These data indicate that the problem is not limited to a single therapeutic barrier but represents a cascade of missed opportunities—ranging from failure to diagnose the condition, through the lack of comprehensive assessment of complications and delayed treatment initiation, to the failure to adequately intensify treatment and provide long-term monitoring.

Therapeutic inertia is of particular importance in this context. In the case of obesity, this may involve delays in the patient or physician recognizing the problem, failure to initiate treatment, continuation of an ineffective strategy despite an unsatisfactory response, and delays in escalating care to pharmacotherapy or metabolic/bariatric surgery. Busetto et al. noted that the accumulation of such delays can result in prolonged exposure to excess adiposity and lead to missed opportunities to prevent the development of T2D, MASLD/MASH, heart failure, or obstructive sleep apnea [132]. The authors also highlight the significant role of obesity-related stigma and the persistent belief that the condition can be effectively addressed primarily by “eating less and increasing physical activity,” which may contribute to delaying more effective interventions [132].

Merely initiating therapy does not resolve the problem. Real-world data indicate significant limitations regarding long-term treatment persistence. An analysis of 26,522 individuals starting anti-obesity pharmacotherapy found that, after six months, treatment was continued by 41.8% of patients receiving liraglutide 3.0 mg, 27.3% using phentermine/topiramate, and 18.1% receiving naltrexone/bupropion [133]. Although these data predate the widespread use of the latest incretin-based therapies and should not be directly extrapolated to current treatment, they effectively illustrate an additional dimension of the “implementation gap”: the efficacy of an intervention demonstrated in clinical trials does not guarantee long-term population-level effectiveness without appropriate monitoring, adherence support, and strategies to prevent disease recurrence.

A contemporary challenge, therefore, lies not only in the availability of guidelines but also in translating them into a sequence of clinical decisions that are feasible within daily practice. The current *Standards of Care in Overweight and Obesity 2026* explicitly recommend the development of clinical practice protocols to support the assessment, staging, and treatment of obesity, while identifying time constraints during consultations as a significant barrier to proper care [134]. Notably, there is evidence that even relatively simple solutions for organizing the clinical process can alter practice: in one study, the automated calculation of BMI within electronic health records was associated with an increase in the documentation of obesity from 31% to 71% and the documentation of interventions from 35% to 59% [135]. A similar philosophy of translating extensive guidelines into simpler clinical pathways was applied in the ESH MASTERplan for hypertension and the CARDIOLIPID Plan for dyslipidemia [136,137]. In the broader context of guideline implementation, the importance of simplification, integration into daily workflows, and decision-support tools is also highlighted as a means of narrowing the evidence-to-practice gap [138].

The ADIPO-CARE Plan was proposed precisely to address this gap. It is not intended to replace existing guidelines or establish new diagnostic criteria or treatment thresholds, but rather to organize available recommendations into a repeatable, sequential management pathway. The ADIPO component systematizes the assessment of adiposity, the disease phenotype, associated complications, and risk factors, leading to the definition of individualized treatment goals; meanwhile, the CARE component structures the implementation of lifestyle interventions and pharmacotherapy, the assessment of treatment response, the timely optimization of strategies, and long-term monitoring of efficacy. Thus, the framework has the potential to mitigate both diagnostic and therapeutic inertia while reducing the fragmentation of care. Its value lies not in adding yet another layer of recommendations to the already complex landscape of obesity guidelines, but in attempting to narrow the gap between what current knowledge dictates should be done for a patient with obesity and what is actually performed in clinical practice. However, the efficacy of the ADIPO-CARE Plan in achieving this objective remains to be evaluated in future implementation and real-world studies.

## 5. Limitations and Future Directions

Several limitations of the ADIPO-CARE Plan should be noted. First, the framework represents an expert-proposed implementation model integrating current scientific evidence and guidelines, rather than a formally validated clinical decision support tool. Its individual components rely on evidence of varying strength, and the proposed management sequence has not yet been prospectively evaluated. Second, certain elements of obesity phenotyping—particularly the use of phenotypes to tailor pharmacotherapy—remain a rapidly evolving field requiring further validation. Third, the feasibility of implementing all components of the ADIPO-CARE Plan may vary across healthcare systems, depending on access to multidisciplinary care, body composition assessment, pharmacotherapy, metabolic/bariatric surgery, and long-term follow-up. Consequently, the model should complement, rather than replace, individual clinical assessment and established guidelines. Future prospective and implementation studies should evaluate whether the ADIPO-CARE Plan enhances diagnostic completeness, complication detection, the appropriate initiation and intensification of therapy, treatment adherence and persistence, and patient-relevant clinical outcomes.

Importantly, no prospective validation study has yet demonstrated that implementing the ADIPO-CARE Plan improves clinical decision-making or patient outcomes compared with standard care.

## 6. Conclusions

The ADIPO-CARE Plan proposes a shift in emphasis from an approach focused primarily on body weight to a model centred on phenotype, risk, complications, and long-term health benefits. The ADIPO stage systematises clinical assessment—from confirming and quantifying adiposity, through determining the behavioural and metabolic phenotype, identifying organ complications, and profiling global cardiometabolic risk, to establishing individualised treatment goals that extend beyond the percentage of weight loss alone. The CARE stage organises treatment and long-term follow-up—from comprehensive lifestyle intervention, through the rational use of anti-obesity pharmacotherapy and systematic response-guided treatment optimisation, to ongoing evaluation of effectiveness, safety, adherence, and relapse prevention over a multiyear perspective. Such a model may systematise clinical decision-making by physicians of different specialities caring for patients with obesity and integrate nutritional therapy with other elements of contemporary, personalised obesity management, in the spirit of precision medicine described in the current literature.

## Figures and Tables

**Table 1:** Comparison of established obesity frameworks and the proposed ADIPO-CARE Plan: principal focus, clinical application, and potential added value.

Framework	Principal Focus	Clinical Application/Characteristic Features	Relationship to ADIPO-CARE Plan
EOSS [10]	Clinical staging	Stages obesity-related medical, mental-health, and functional burden beyond BMI.	ADIPO-CARE Plan incorporates complication and functional assessment into a wider assessment-to-treatment pathway.
5As [12]	Patient-centred clinical interaction	Ask, Assess, Advise, Agree, Assist; supports communication, shared decisions, and continuing support.	ADIPO-CARE Plan offers a complementary, more explicitly sequenced set of clinical assessment and treatment tasks; it does not replace the 5As communication approach.
COTA (AACE/ACE) [14]	Complications-centric treatment	Uses the presence and severity of obesity-related complications to inform treatment intensity and goals.	ADIPO-CARE Plan retains complication-guided decision-making while organizing subsequent treatment selection, response review, and follow-up.
ABCD/EASO [7,15,16,17]	Adiposity-based disease assessment and comprehensive care	Integrates adiposity, health risk, complications, and individualized multidisciplinary management.	ADIPO-CARE Plan translates overlapping recommendations into a concise two-stage, nine-step workflow; it does not claim broader clinical coverage than EASO guidance.
Lancet Commission (2025) [6]	Definition and diagnosis	Distinguishes clinical from preclinical obesity on the basis of excess adiposity and objective organ or tissue dysfunction.	ADIPO-CARE Plan situates diagnostic assessment within a proposed longitudinal care pathway; it does not redefine clinical obesity.
ADIPO-CARE Plan (proposed)	Integrated clinical workflow	Five ADIPO assessment steps followed by four CARE treatment and follow-up steps.	Potential contribution: a single mnemonic pathway linking assessment, treatment individualization, response-guided optimization, and longitudinal care; not yet prospectively validated.

ABCD—adiposity-based chronic disease; AACE/ACE—American Association of Clinical Endocrinologists/American College of Endocrinology; BMI—body mass index; COTA—Complications-Centric Obesity Treatment Algorithm; EASO—European Association for the Study of Obesity; EOSS—Edmonton Obesity Staging System; 5As—Aks, Assess, Advise, Agree, Asist.

**Table 2:** The cut-off points for waist circumference (WC), waist-to-hip ratio (WHR) by sex and population group.

Source/Criterion [Ref]	Sex	Cut-Off	Risk Interpretation
**Waist Circumference (WC)**			
WHO (2008/2011) [21]	Men	>94 cm	Increased risk of metabolic complications
	Women	>80 cm	
	Men	>102 cm	Substantially increased risk of metabolic complications
	Women	>88 cm	
NCEP ATP III (2001) [22]	Men	≥102 cm	Abdominal obesity—component criterion for metabolic syndrome
	Women	≥88 cm	
IDF—Europids (2006) [23]	Men	≥94 cm	Central obesity
	Women	≥80 cm	
IDF—South Asians, Chinese (2006) [23]	Men	≥90 cm	Central obesity
	Women	≥80 cm	
IDF—Japanese (2006) [23]	Men	≥85 cm	Central obesity
	Women	≥90 cm	
IDF—Central/South America, Sub-Saharan Africa, Middle East [23]	Use South Asian/European values *		Pending population-specific data
**Waist-to-Hip Ratio (WHR)**			
WHO (2008/2011) [23]	Men	≥0.90	Substantially increased risk of metabolic complications
	Women	≥0.85	

*—For populations without established ethnicity-specific waist circumference cutoffs, the IDF recommended the provisional use of South Asian or European thresholds until population-specific data become available; NCEP ATP—National Cholesterol Education Program Adult Treatment Panel; IDF—International Diabetes Federation.

**Table 3:** Metabolic phenotypes according to body weight, body composition, and metabolic health. Based on information from [34]. Metabolic abnormalities refer to the metabolic syndrome defining criteria. The classification presented here is descriptive in nature; the criteria used to define metabolic health vary across studies and do not constitute a uniform, universally validated diagnostic system.

Parameter	MHNW (Metabolically Healthy Normal Weight)	MUNW (Metabolically Unhealthy Normal Weight)	MHO (Metabolically Healthy Obese)	MUO (Metabolically Unhealthy Obese)
**WC**	Normal	Normal/increased	Normal	Increased
**BMI**	18.5–24.9	18.5–24.9	>25	>25
**Truncal body** **fat content**	Low	High	Low	High
**Level of** **lean body mass**	–	–	High	–
**Metabolic** **disturbances**	Absent	Present	Absent	Present
**Cardiometabolic** **risk**	↔	↑	↑	↑

BMI—body mass index; WC—waist circumference.

**Table 4:** Selected treatment goals in obesity management (based on information from [60]).

Obesity-Related Complication	Clinical Goal: Weight Reduction by	Therapeutic Goal: Expected Health Improvement
Prediabetes	≥10%	Prevention of progression to type 2 diabetes
Type 2 diabetes	≥5–15%	(1) Reduction in HbA1c (2) Reduction in number and/or doses of glucose-lowering medications (3) Diabetes remission, especially if of recent onset
Atherogenic dyslipidaemia	≥5–15%	Reduction in TG and non-HDL-C, with an increase in HDL-C
Arterial hypertension	≥5–15%	(1) Reduction in systolic and diastolic blood pressure (2) Reduction in number and/or doses of antihypertensive medications
MASLD	≥5%	Reduction in hepatic steatosis
MASH	≥10–40%	Reduction in inflammation and inhibition of fibrosis
PMOS	≥5–15%	(1) Restoration of ovulation (2) Regulation of the menstrual cycle (3) Reduction in hirsutism and acne severity (4) Improved insulin sensitivity (5) Reduction in blood androgen concentration
Female infertility	≥10%	(1) Improved ovulation (2) Increased chance of pregnancy
Male hypogonadism	≥5–10%	Increase in blood testosterone concentration
Obstructive sleep apnoea	≥7–11%	Reduction in the apnoea–hypopnoea index
Asthma	≥7–8%	Increase in FEV1
Osteoarthritis	≥10%	Reduction in symptom severity
Stress urinary incontinence	≥5–10%	Reduction in incontinence frequency
Gastro-oesophageal reflux	≥10%	Reduction in symptom severity

FEV1—forced expiratory volume in one second; HbA1c—glycated haemoglobin; HDL-C—high-density lipoprotein cholesterol; MASLD—metabolic dysfunction-associated steatotic liver disease; MASH—metabolic dysfunction-associated steatohepatitis; PMOS—polyendocrine metabolic ovarian syndrome; TG—triglycerides.

**Table 5:** Evidence from clinical trials supporting the selection of specific agents in the pharmacological treatment of obesity (based on [16]).

Treatment Goal	Subgroup	Endpoint	Naltrexone- Bupropion	Liraglutide	Phentermine- Topiramate	Semaglutide	Tirzepatide
**Weight** **Reduction**	**No complications**	**Total body weight loss (%)**					
		*At end of trial*	4.8 ^a^	4.2 ^a^	8.8 ^a^	8.7 ^a^	16.5 ^a^
		*At week 52*	4.8 ^a^	7.0 ^a^	8.8 ^a^	10.7 ^a^	14.8 ^a^
		*At weeks 53–104*	4.8 ^a^	4.3 ^a^	8.8 ^a^	10.1 ^a^	16.5 ^a^
**Treatment of** **Obesity** **Complications**	***Complications resulting from excess fat mass***						
	Sleep apnea	*Apnea remission (OR)*	NA	NA	NA	NA	2.9 ^a^
	Knee osteoarthritis	*Improvement (WMD)*	NA	NA	NA	−8.6 ^a^	NA
	***Complications resulting from adipose tissue dysfunction***						
	Prediabetes	*Restoration of normoglycemia (OR)*	NA	3.25 ^a^	NA	19.6 ^a^	8.3 ^a^
	Type 2 diabetes	*Diabetes remission (OR)*	2.3 ^a^	6.8 ^a^	NA	12.3 ^a^	15.6 ^a^
	Cardiovascular disease	*MACE occurrence (OR)*	0.88	NA	NA	0.78 ^a^	NA
	Heart failure	*Hospitalization (OR)*	NA	NA	NA	0.23 ^a^	0.45 ^a^
	MASLD	*MASH remission (OR)*	NA	NA	NA	2.0	11.8 ^a^

Improvement in knee osteoarthritis is defined as reduced pain and/or improved physical function, as assessed using a validated questionnaire. MACE: major atherosclerotic cardiovascular events; MASH: metabolic dysfunction-associated steatohepatitis; MASLD: metabolic dysfunction-associated steatotic liver disease; NA: no available data; OR: odds ratio after adjusting for placebo; WMD: weighted mean difference after adjusting for placebo. ^a^ Statistically significant result (*p* < 0.05).

## Data Availability

No new data were created or analyzed in this study. Data Sharing is not applicable to this article.

## Abbreviations

The following abbreviations are used in this manuscript:

AACE	American Association of Clinical Endocrinology
ABCD	adiposity-based chronic disease
ACC	American College of Cardiology
ACE	American College of Endocrinology
ACR	albumin-to-creatinine ratio
ACTION-IO	Awareness, Care, and Treatment In Obesity maNagement—International Observation
ADA	American Diabetes Association
AHA	American Heart Association
ALT	alanine aminotransferase
ASCVD	atherosclerotic cardiovascular disease
ASMBS	American Society for Metabolic and Bariatric Surgery
ASN	American Society of Nephrology
AST	aspartate aminotransferase
BEDS-7	Binge Eating Disorder Screener-7
BIA	bioelectrical impedance analysis
BMI	body mass index
BNP	B-type natriuretic peptide
CKD	chronic kidney disease
CKD-EPI	Chronic Kidney Disease Epidemiology Collaboration
CKM	cardiovascular-kidney-metabolic syndrome
CLI	comprehensive lifestyle intervention
COTA	Complications-Centric Obesity Treatment Algorithm
CT	computed tomography
CV	cardiovascular
DASH	Dietary Approaches to Stop Hypertension
DXA	dual-energy X-ray absorptiometry
EASD	European Association for the Study of Diabetes
EASL	European Association for the Study of the Liver
EASO	European Association for the Study of Obesity
ECG	electrocardiogram
EOSS	Edmonton Obesity Staging System
EPIC	European Prospective Investigation into Cancer and Nutrition
ESC	European Society of Cardiology
ESH	European Society of Hypertension
ESPEN	European Society for Clinical Nutrition and Metabolism
FEV1	forced expiratory volume in 1 s
FIB-4	Fibrosis-4 Index
FSGS	focal segmental glomerulosclerosis
FSH	follicle-stimulating hormone
GAD-7	Generalized Anxiety Disorder-7
GFR	glomerular filtration rate
GGT	gamma-glutamyl transferase
GIP	glucose-dependent insulinotropic polypeptide
GLP-1	glucagon-like peptide-1
GLP-1RA	glucagon-like peptide-1 receptor agonist
HADS	Hospital Anxiety and Depression Scale
HbA1c	glycated haemoglobin
HDL	high-density lipoprotein
HDL-C	high-density lipoprotein cholesterol
HF	heart failure
HFpEF	heart failure with preserved ejection fraction
IDF	International Diabetes Federation
IFSO	International Federation for the Surgery of Obesity and Metabolic Disorders
LDL-C	low-density lipoprotein cholesterol
LH	luteinizing hormone
LVEF	left ventricular ejection fraction
MACE	major adverse cardiovascular events
MASH	metabolic dysfunction-associated steatohepatitis
MASLD	metabolic dysfunction-associated steatotic liver disease
MBS	metabolic/bariatric surgery
MHNW	metabolically healthy normal weight
MHO	metabolically healthy overweight/obesity
MRI	magnetic resonance imaging
MUNW	metabolically unhealthy normal weight
MUO	metabolically unhealthy overweight/obesity
NAION	non-arteritic anterior ischemic optic neuropathy
NCEP	National Cholesterol Education Program
NHANES	National Health and Nutrition Examination Survey
NT-proBNP	Nterminal pro-B-type natriuretic peptide
ORG	obesity-related glomerulopathy
OSA	obstructive sleep apnoea
PCOS	polycystic ovary syndrome
PHQ-9	Patient Health Questionnaire-9
PMOS	polyendocrine metabolic ovarian syndrome
QoL	quality of life
SCORE2	Systematic Coronary Risk Evaluation 2
SCORE2-Diabetes	Systematic Coronary Risk Evaluation 2-Diabetes
SCORE2-OP	Systematic Coronary Risk Evaluation 2-Older Persons
SHBG	sex hormone-binding globulin
STOP-BANG	Snoring, Tiredness, Observed apnoea, high blood Pressure, Body mass index, Age, Neck circumference, and Gender
T2D	type 2 diabetes
TBWL	total body weight loss
TC	total cholesterol
TFEQ-21	Three-Factor Eating Questionnaire-21
TG	triglycerides
UDCA	ursodeoxycholic acid
VCTE	vibration-controlled transient elastography
VLCD	very-low-calorie diet
WC	waist circumference
WHO	World Health Organization
WHR	waist-to-hip ratio
WHtR	waist-to-height ratio
WMD	weighted mean difference
